# Diversity and disparity of sparassodonts (Metatheria) reveal non-analogue nature of ancient South American mammalian carnivore guilds

**DOI:** 10.1098/rspb.2017.2012

**Published:** 2018-01-03

**Authors:** Darin A. Croft, Russell K. Engelman, Tatiana Dolgushina, Gina Wesley

**Affiliations:** 1 Department of Anatomy, Case Western Reserve University, Cleveland, OH, USA; 2 Department of Biology, Case Western Reserve University, Cleveland, OH, USA; 3 School of Writing, Literature and Film, Oregon State University, Corvallis, OR, USA; 4 Department of Biology, Montgomery College, Rockville, MD, USA

**Keywords:** Carnivora, dentition, marsupial, palaeobiology, palaeoecology, Sparassodonta

## Abstract

This study investigates whether terrestrial mammalian carnivore guilds of ancient South America, which developed in relative isolation, were similar to those of other continents. We do so through analyses of clade diversification, ecomorphology and guild structure in the Sparassodonta, metatherians that were the predominant mammalian carnivores of pre-Pleistocene South America. Body mass and 16 characters of the dentition are used to quantify morphological diversity (disparity) in sparassodonts and to compare them to extant marsupial and placental carnivores and extinct North American carnivoramorphans. We also compare trophic diversity of the Early Miocene terrestrial carnivore guild of Santa Cruz, Argentina to that of 14 modern and fossil guilds from other continents. We find that sparassodonts had comparatively low ecomorphological disparity throughout their history and that South American carnivore palaeoguilds, as represented by that of Santa Cruz, Argentina, were unlike modern or fossil carnivore guilds of other continents in their lack of mesocarnivores and hypocarnivores. Our results add to a growing body of evidence highlighting non-analogue aspects of extinct South American mammals and illustrate the dramatic effects that historical contingency can have on the evolution of mammalian palaeocommunities.

## 1. Introduction

The evolution of mammals during South America's protracted Cenozoic geographical isolation is well documented [1–3]. Nevertheless, few studies have attempted to examine how such ‘Splendid Isolation’ may have affected the structure of mammalian ecological communities. Herein, we quantify and analyse the taxonomic diversity and morphological disparity of sparassodont metatherians, the predominant carnivorous land mammals of ancient South America, in order to characterize their evolutionary history and examine dietary resource partitioning among members of the terrestrial carnivore guild. Sparassodonts represent a radiation of mammals into the carnivore/predator niche independent of those on other continents [4]. Thus, their fossil record provides an opportunity to test whether patterns of clade evolution and niche partitioning in carnivoramorphans primarily reflect ecological factors affecting all carnivorous mammals or morphological adaptations unique to Carnivora.

**Figure 1.**
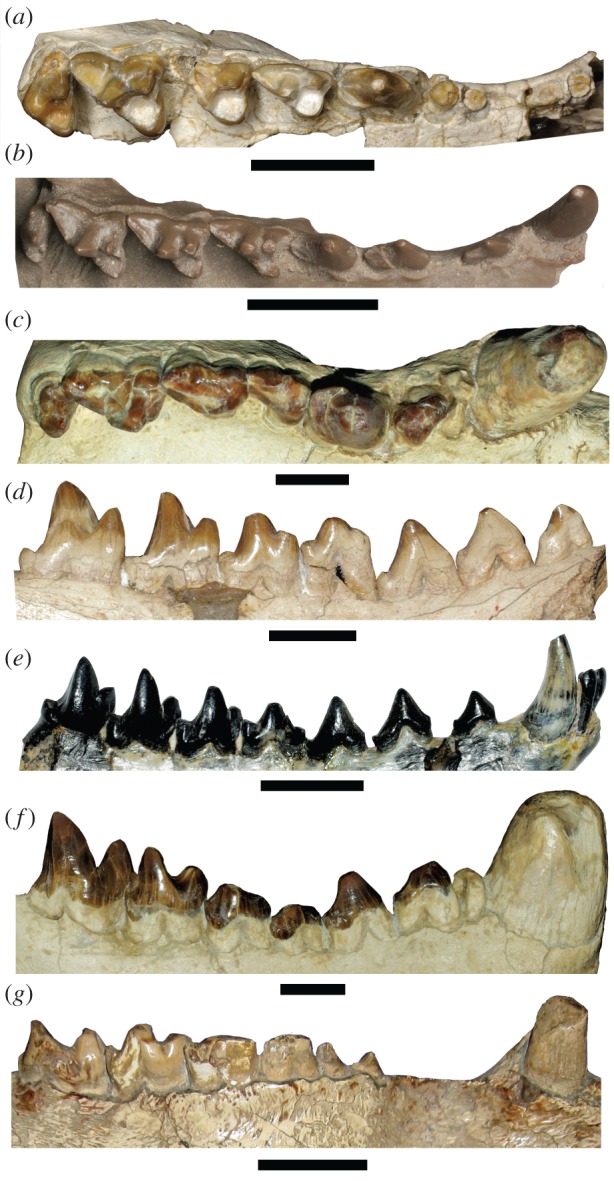
Representative sparassodont upper dentitions in occlusal view (*a–c*) and lower dentitions in lingual (*d*) and buccal (*e–g*) views, anterior to right. (*a*) *Hondadelphys fieldsi*, left P1-P2 roots and P3-M4 (reversed), UCMP 37960; (*b*) *Sipalocyon gracilis,* left C-M4, cast of YPM-PU 15373; (*c*) *Arctodictis sinclairi*, left C-M4 (reversed), MLP 85-VII-3–1; (*d*) *Pseudothylacynus rectus*, left p1-m4, MACN-A 52-369; (*e*) *Sipalocyon gracilis*, left i1-m4 (reversed), MACN-A 691; (*f*) *Arctodictis sinclairi*, right c-m4, MLP 85-VII-3-1; (*g*) *Thylacosmilus atrox*, left c-m4 (reversed), FMNH P14344. Scale bars equal 1 cm. For collection abbreviations, see the electronic supplementary material, table S5. (Online version in colour.)

Sparassodonts are organized into five family-level groups: Borhyaenidae, Proborhyaenidae, Thylacosmilidae, Hathliacynidae and the monotypic Hondadelphidae, the first three of which comprise Borhyaenoidea [5–7]. They ranged in size from less than 1 kg to approximately 150 kg, and their fossil record extends from the early Cenozoic to the Pliocene (electronic supplementary material, table S1). Although sparassodonts as a group were clearly carnivorous (figure 1), opinions have varied regarding their roles in ancient South American mammal communities. Some species have universally been regarded as meat or meat/bone specialists [8,9], whereas others have been characterized as more omnivorous based on qualitative (rather than quantitative) comparisons [2,10,11]. Opinions are similarly divergent regarding the taxonomic diversity and therefore ecological structure of South America's mammalian carnivore guild prior to the Great American Biotic Interchange [12–14].

Our study, which analyses the functional morphology of the entire sparassodont dentition, aims to determine how the trophic structure of a terrestrial carnivore guild from the middle Cenozoic of South America compares to modern and fossil terrestrial carnivore guilds from other continents. We also address several other fundamental questions about carnivore evolution, palaeoecology and guild structure in South America including: (i) did the diversification of sparassodonts in South America resemble that of carnivoramorphans in North America? (ii) how did the late Cenozoic decline of sparassodonts affect the group's disparity? and (iii) how do diversity and disparity of sparassodonts compare to Cenozoic North American carnivoramorphans and modern carnivorans as a whole?

## 2. Material and methods

### (a) Terminology

‘Diversity’ (without any qualifier) refers to taxonomic diversity (richness) and ‘disparity’ refers to morphological diversity [15] or functional richness [16]. We measure diversity using operational taxonomic units (OTUs) and disparity using occupied morphospace. ‘Carnivorous’ refers to animals that feed on vertebrates (not simply secondary consumers). ‘Omnivorous’ refers to animals that consume significant plant material and/or invertebrates in addition to vertebrates.

### (b) Sparassodont data

OTUs are genera or purported genera, primarily following Forasiepi [5]. Additional details are provided in the electronic supplementary material, table S4. To describe sparassodont dental ecomorphology, we scored all OTUs for 16 characters used by several previous studies [17–19] to quantify carnivoramorphan and ‘creodont’ dental ecomorphology (electronic supplementary material, tables S6–S7). M3 and m4 were considered the carnassials in sparassodonts, as they are typically the largest and least-worn slicing teeth and are thought to be the closest functional analogues of ‘creodont’ and carnivoran carnassials (e.g. [19,20]). Relative grinding area (Character no. 16) was scored following Werdelin & Wesley-Hunt [17] but with one bin subdivided into order to better distinguish extant mesocarnivores and hypocarnivores. Body mass (Character no. 17) was scored following Wesley-Hunt [19] but with the smallest body size state divided into two; OTUs were coded using body mass ranges in the electronic supplementary material, table S1, and scored for the larger state in cases where the body mass spanned two categories. Scoring was based on original material where possible (electronic supplementary material, table S5). Owing to incomplete fossil preservation, most OTUs could not be scored for at least some characters. In some cases, character states were scored based on homologous or analogous teeth or morphologically similar OTUs (noted in the electronic supplementary material, table S7).

### (c) Non-sparassodont data

We coded nine additional carnivorous metatherian genera for comparative purposes: three extant Australian dasyuromorphians and six extinct South American didelphimorphians (electronic supplementary material, table S1). The former may be the closest extant ecomorphological analogues for sparassodonts, whereas the latter coexisted with sparassodonts during the late Neogene and have been suggested to have occupied similar niches and/or competitively replaced some species [10,21]. Metatherian codings were combined with the extant carnivoran dataset of Werdelin & Wesley-Hunt [17], which includes approximately 85% of modern carnivoran species, and the North American Cenozoic carnivoramorphan dataset of Wesley-Hunt [19], which was recoded to be congruent with the present study.

### (d) Time bins

For analyses of diversity and disparity through time, sparassodonts were allocated to 2-million-year time bins based on the South American Land Mammal Age(s) (SALMAs) or informal equivalents in which the taxon has been recorded (electronic supplementary material, table S8). These bins (intervals) were used rather than absolute ages because the ages of most South American fossil sites are not known with greater precision.

### (e) Carnivore guilds

Taxonomic lists of non-volant terrestrial carnivore guilds were compiled from the literature for the late Early Miocene site of Santa Cruz, Argentina [22,23] and three modern ecosystems: the lowland rainforest of Malaysia, the savannah-woodland of Serengeti National Park, Tanzania, and the temperate coniferous forest of Yellowstone National Park, USA (electronic supplementary material, table S9). Santa Cruz was chosen as representative of a South American fossil carnivore guild because it is the most diverse guild presently known, and the palaeobiology of many species has been studied in detail. The three modern sites are those used by Van Valkenburgh in her classic studies of large carnivore guilds [24,25]; small carnivorans were added based on other sources. Modern carnivorans were categorized as hypercarnivorous, mesocarnivorous or hypocarnivorous based primarily on Van Valkenburgh [25] (large species) and Friscia *et al.* [26] (small species). A comparative set of 11 Northern Hemisphere fossil carnivore guilds (including ‘creodonts’, mesonychians, carnivoramorphans and other groups) were compiled from Morlo *et al.* [27] and the primary literature (electronic supplementary material, table S9).

### (f) Analytical methods

Analytical methods for calculating disparity follow Werdelin & Lewis [28] and Werdelin & Wesley-Hunt [18]. Disparity (occupied morphospace [29]) was calculated as the convex hull area encompassing the taxa on a bivariate plot of the first two axes of a correspondence analysis. All OTUs (sparassodonts, dasyuromorphians, didelphimorphians, North American Cenozoic carnivoramorphans and modern carnivorans) were included in a single analysis to obtain individual scores that were subsequently used to determine occupied morphospace. Morphospace analyses were performed in PAST v. 3.10 for Mac [30], which uses column average substitution to accommodate missing data. Other statistical analyses and data visualization were conducted in JMP Pro^®^ 13.0 for Mac [31].

## 3. Results

### (a) Diversification and decline of sparassodonts

**Figure 2.**
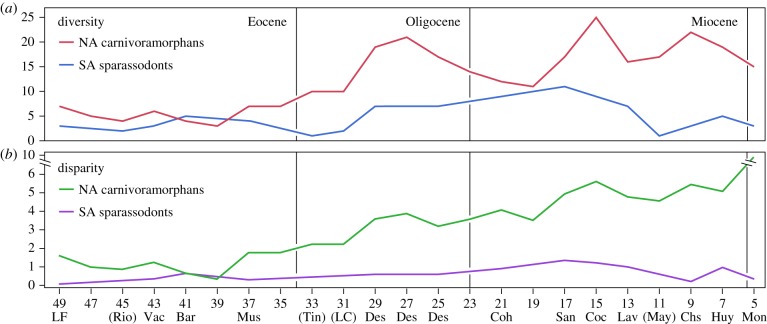
Graphs of diversity (*a*) and disparity (*b*) through time for South American (SA) sparassodonts and North American (NA) carnivoramorphans. Only Eocene to Early Pliocene bins are shown because sparassodont disparity cannot be calculated beyond this range (less than 3 OTUs). *X*-axis values are midpoints of 2-million-year time bins. Bins represented in the South American fossil record are labelled with the corresponding interval (see the electronic supplementary material, table S1 for abbreviations); parentheses indicate bins with fewer than three sparassodont OTUs (for which disparity cannot be calculated). (Online version in colour.)

Sparassodont disparity is positively correlated with diversity (Spearman's *ρ* = 0.8350 excluding intervals with <3 OTUs; *p* < 0.0002), and maximal disparity coincides with maximal diversity during the late Early Miocene (Santacrucian interval; figure 2). Disparity increases from the Early to Middle Eocene (Barrancan interval), drops during the Late Eocene (Mustersan interval) and gradually increases up to the late Early Miocene peak. It decreases gradually during the Middle Miocene, drops during the early-Late Miocene, subsequently rebounds and then drops again by the end of the Miocene (see also Discussion). Diversity shows a similar but exaggerated trend, particularly when intervals with less than three OTUs are ignored (i.e. those for which disparity cannot be calculated). Disparity relative to diversity is highest during the Late Miocene (Huayquerian interval), which plots as an outlier relative to other points based on Mahalanobis and jackknife distances at *α* = 0.05 (but not *α* = 0.01).

### (b) Diversification of carnivoramorphans

Carnivoramorphan disparity is also positively correlated with diversity (Spearman's *ρ* = 0. 8922; *p* < 0.0001). Disparity decreases after the Early Eocene and reaches a low point during the Late Eocene (40–38 Ma) but generally increases throughout the remainder of the Cenozoic. Dips in disparity occur during the Oligocene (30–26 Ma), Early Miocene (20–18 Ma) and Middle Miocene (14–10 Ma), and disparity rises steeply in the Late Miocene (after 6 Ma). Diversity outpaces disparity 29–27 Ma but decreases during the Late Oligocene and Early Miocene (26–20 ma), while disparity remains generally constant. Disparity and diversity show congruent patterns in the Early to Middle Miocene (20–12 Ma), but a decrease in disparity coincides with an increase in diversity in the late Middle Miocene (12–10 Ma), and the opposite occurs during the Late Miocene (after 6 Ma). This final interval (6–4 Ma) plots as an outlier relative to other points based on Mahalanobis and jackknife distances (*p* < 0.01).

### (c) Sparassodonts and North American carnivoramorphans compared

Carnivoramorphan diversity exceeds that of sparassodonts throughout the Cenozoic except during the late Middle Eocene (42–40 Ma; Barrancan interval), when five OTUs are recorded in South America and only four in North America. In nearly every other interval in which a direct comparison is possible, carnivoramorphan diversity is at least twice that of sparassodonts. The only notable exception to this pattern is the Early Miocene (Colhuehuapian and Santacrucian intervals), when carnivoramorphan diversity is only approximately 1.3–1.5× that of sparassodonts.

Disparity displays a similar pattern to diversity. Carnivoramorphan disparity exceeds that of sparassodonts throughout the Cenozoic, though values are nearly identical during the late Middle Eocene (Barrancan interval; 0.66163 versus 0.64365, respectively). Carnivoramorphan disparity is 2–2.3× that of sparassodonts prior to this time and generally approximately 4–7× that of sparassodonts thereafter. Much greater discrepancies (approx. 26–29×) correspond to the two late Cenozoic dips in sparassodont disparity (Chasicoan and Montehermosan intervals).

Total disparity of Cenozoic North American carnivoramorphans (14.046; *n* = 95) is approximately 85% of that of extant carnivoramorphans worldwide (16.536; *n* = 216). Total disparity of sparassodonts (2.2386; *n* = 41) is approximately 15% that of carnivoramorphans today and throughout the Cenozoic.

### (d) Morphospace occupation (disparity)

**Figure 3.**
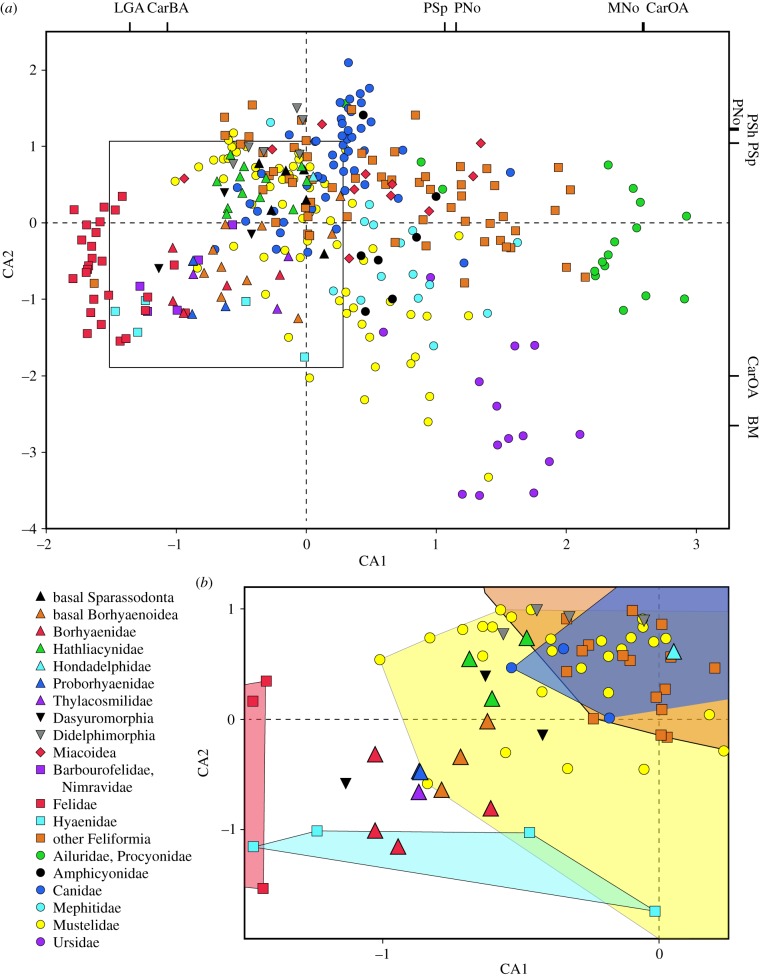
(*a*) Plot of all OTUs (sparassodonts, dasyuromorphians, didelphimorphians, North American Cenozoic carnivoramorphans and modern carnivorans) on first two canonical axes (CAs) of an analysis of 16 functional characters of the dentition and body mass. ‘Other Feliformia’ includes Eupleridae, Herpestidae, Nandiniidae, Prionodontidae and Viverridae. Characters (Ch.) with positive or negative loadings greater than 1.0 are plotted on the corresponding CA. See the electronic supplementary material, tables S10 and S11, for character loadings and scores for individual taxa, respectively. (*b*) Inset box of (*a*) showing only modern taxa with CA1 values < 0.5 and CA2 values < 1.0 and sparassodonts coded for 12 or more characters. Shaded areas represent entire morphospace occupied by group in (*a*). Abbreviations: BM, body mass (Ch. 17); CarBA, lower carnassial buccal angle (Ch. 11); CarOA, upper carnassial occlusal angle (Ch. 10); LGA, lower grinding area (Ch. 16); MNo, number of upper molars (Ch. 14); PNo, number of upper premolars (Ch. 3); PSh, upper premolar shape (Ch. 4); PSp, upper premolar spacing (Ch. 5).

The first two canonical axes (CAs) encompass 55.3% of the variation among taxa (figure 3*a*). The distributions of taxa and characters strongly resemble the results of Werdelin & Wesley-Hunt [17]; the *x*-axis is inversely correlated with carnivory (hypercarnivores towards the left, hypocarnivores towards the right), whereas the *y*-axis correlates negatively with body mass and upper carnassial occlusal angle, and positively with the number of upper premolars anterior to the carnassial and shape of the largest upper premolar anterior to the carnassial.

Nearly all metatherians plot negatively on CA1; exceptions include *Pseudolycopsis*, IGM 251108, *Stylocynus* and *Hondadelphys*, which have low positive values (approx. less than or equal to 0.25), and *Nemolestes* and *Notogale*, which have values close to zero (less than 0.005). On CA2, metatherians straddle the origin, well within the range spanned by modern carnivorans. Didelphimorphians and sparassodonts occupy distinct morphospaces in figure 3*a* except for *Thylatheridium*, which is just within hathliacynid morphospace. This agrees with the findings of Zimicz [32].

Total sparassodont disparity (2.0765; *n* = 41) is approximately 12% that of modern carnivorans worldwide (17.468; *n* = 216) and approximately 15% that of Cenozoic North American carnivoramorphans (13.93; *n* = 95). It is only approximately 15% that of modern South American carnivorans (13.681), which are similarly diverse (*n* = 40). Sparassodont disparity is comparable to that of extant canids (2.1477) but less than that of moderately disparate families such as Eupleridae (2.4035) and Procyonidae (2.5734) (see the electronic supplementary material, table S12).

### (e) Sparassodont diets

Sparassodonts scored for all or nearly all (12 out of 13) characters (*n* = 14) plot most closely to modern hypercarnivorous species (figure 3*b*). Most occupy a morphospace between felids and hypercarnivorous canids that overlaps hyaenids and highly carnivorous mustelids including the honey badger (*Mellivora capensis*), African striped weasel (*Poecilogale albinucha*), beech martin (*Martes foina*), wolverine (*Gulo gulo*) and weasels of the genus *Mustela*. *Hondadelphys* is an outlier among sparassodonts, plotting near hypercarnivorous canids and a variety of carnivorous to omnivorous feliforms (herpestids, viverrids and *Nandinia*). Dasyuromorphians largely overlap sparassodonts, occupying positions relative to placental carnivorans similar to those found by Jones [33].

### (f) Carnivore guilds

**Figure 4.**
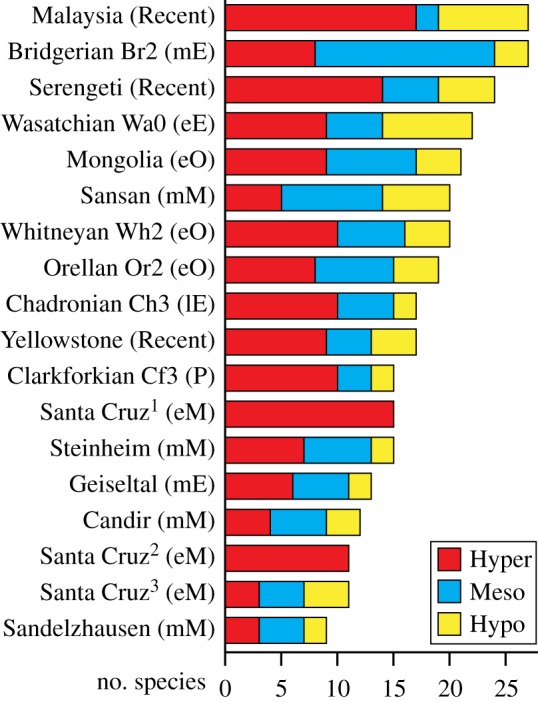
Stacked bar graphs comparing diversity of hypercarnivores (Hyper), mesocarnivores (Meso) and hypocarnivores (Hypo) in the Santa Cruz Formation of Argentina to three modern and 11 fossil terrestrial carnivore guilds, arranged by decreasing number of species. Age abbreviations (in parentheses): e, Early; E, Eocene; l, Late; m, Middle; M, Miocene; O, Oligocene; P, Palaeocene. Superscript numbers for Santa Cruz indicate: 1, diets following Ercoli *et al.* [23], with phorusrhacids (terror birds) included; 2, diets following Ercoli *et al.* [23] and 3, diets following Marshall [10]. See the electronic supplementary material, table S9 for species lists and dietary categories for Santa Cruz, White River Group biozones (Ch3, Or2, Wh2; USA), and modern guilds. Data for other fossil guilds are from table 10.1 of Morlo *et al.* [27] with ‘bone/meat’ species grouped with hypercarnivores, ‘carnivore’ species treated as mesocarnivores, and ‘insectivorous’ species grouped with hypocarnivores. (Online version in colour.)

Disparity of the Santa Cruz carnivore guild (0.74619) is far less than that of the modern carnivore guilds of Malaysia (11.409), the Serengeti (5.5325) and Yellowstone (11.566). This fits with the low trophic diversity inferred for the Santa Cruz carnivore guild; all sparassodonts from the site have been interpreted as hypercarnivores, congruent with their positions in figure 3, whereas only one-half to two-thirds of species in modern and fossil carnivore guilds from other continents are hypercarnivores (figure 4; electronic supplementary material, table S9). If the four Santa Cruz phorusrhacids (terror birds) are included, trophic diversity remains unchanged, as phorusrhacids are also interpreted as hypercarnivores [10,22]. Species diversity of the Santa Cruz carnivore guild is 40–65% that of the modern guilds analysed and 40–75% that of most fossil guilds; with phorusrhacids, this increases to 55–90% compared to modern guilds and 55–100% for most fossil guilds. The number of hypercarnivores at Santa Cruz (15 including phorusrhacids) is comparable to that of Malaysia (*n* = 17) and Serengeti (*n* = 14) but 1.5–5× the number recorded at other fossil sites.

## 4. Discussion

### (a) Completeness of the fossil record

More than one-third of sparassodont character states could not be coded (257 of 697 states; electronic supplementary material, table S7). This reflects the rarity of sparassodont specimens in the fossil record [9,12,13,34] as well as their generally poor preservation (cf. [11,35,36]). The relative contributions of ecology, taphonomy, sampling and other factors to such scarcity remain unresolved, but the sparassodont record should be interpreted with caution, given the large amount of missing data. Hence, we focus our discussion on broad temporal patterns, a well-sampled fossil site (Santa Cruz) and taxa known from relatively complete remains.

### (b) Evolution of the Sparassodonta

Sparassodont diversity and disparity are positively correlated and generally increase from the group's earliest record to its Early Miocene peak. This most closely corresponds to the idealized diversification model C of Foote [37], a pattern in which diversity and disparity are concordant that can result from diffusion in morphospace or adaptive radiation [37]. The Early Oligocene (Tinguirirican) drop in diversity (figure 2) is almost certainly an artefact of the very limited fossil record for this interval, which is only sampled from a few sites [38,39] and has not yielded specimens complete enough to be analysed. Curiously, sparassodonts do not appear to have decreased in diversity or disparity during the Middle Eocene, unlike North American carnivoramorphans, ‘creodonts’ and other groups [40–42]. This could indicate little significant climate/habitat change in Patagonia during this interval, as has been documented for the later Palaeogene [43], or be an artefact of comparatively poor sampling of earlier intervals.

The relatively high disparity recorded before the extinction of the sparassodonts (figure 2) corresponds most closely to Foote's model G of clade decline in which ‘selection against morphological intermediates causes an increase in morphological variance, analogous to disruptive selection within populations' [37]. In other words, sparassodonts remained broadly separated in morphospace despite decreasing Late Cenozoic diversity (electronic supplementary material, figure S1). Based on our analysis, Late Miocene sparassodont disparity was greater than Late Oligocene disparity, and we find no support for a protracted decline after the Deseadan interval (Late Oligocene), as postulated by Marshall [10]. A decrease in disparity may have commenced after the Early Miocene, but a post-Middle Miocene or even post-Miocene decline is more likely given sampling differences among intervals; the early Late Miocene represents a large gap in knowledge, with only one well-sampled site of Chasicoan age and no sparassodonts yet discovered from the few known Mayoan sites [5]. Sparassodont diversity broadly parallels disparity except that diversity is greater during the Late Oligocene than the Late Miocene.

It has been suggested that constraints on molar morphology related to tooth replacement in metatherians may have limited the diversity and disparity of carnivorous forms [44]. Our analysis of the entire dentition supports this conclusion. At nearly every point throughout the Cenozoic, the terrestrial carnivore guild of South America was less disparate than that of North America (figure 2). Moreover, sparassodonts as a clade are less disparate than South American carnivorans today (electronic supplementary material, table S11), despite comparable diversity (41 versus 40 OTUs, respectively). Sparassodonts are also less disparate than euplerids, which include a mere seven species that evolved to occupy hypocarnivore, mesocarnivore and hypercarnivore niches in Madagascar in fewer than 25 Ma [17,45].

The reasons for the decline and extinction of the Sparassodonta remain unknown. Competition with North American carnivorans appears to be an unsatisfactory explanation [20,46], as does displacement by didelphoids [6,32]. A recent study [47] concluded that the primary factor may have been non-competitive ecological interactions, perhaps coupled with climate, though precisely what those ecological interactions may have been is unclear.

### (c) Ecological communities

Comparing the terrestrial carnivore guild of Santa Cruz, Argentina to selected modern and fossil guilds from other continents yields two noteworthy observations (figure 4). First, diversity is low: the guild includes fewer mammal species than all three modern carnivore guilds and all but one fossil guild. Second, the distribution of species among trophic categories is unlike any other guild. If this accurately represents the Santa Cruz carnivore guild, it presents an ecological anomaly: why are there no mesocarnivores or hypocarnivores?

One potential explanation is sampling: sparassodonts filled these niches but simply were not preserved in the fossil record. This is a reasonable explanation for most fossil sites, but the Santa Cruz Fauna derives from what is probably South America's most productive fossil mammal-producing formation and is considered to faithfully represent its ancient carnivore guild [9,23]. In fact, the abundance of Santa Cruz specimens, combined with the formation's broad geographical extent and stratigraphic thickness, has resulted in the opposite problem for most mammal groups: a plethora of invalid junior synonyms and an overestimation of diversity [35,48,49]. Thus, it is unlikely that a significant portion of the Santa Cruz carnivore guild remains unsampled.

Alternatively, Santa Cruz may not represent a typical Cenozoic South American ecological community. This relatively high-latitude site (approx. 51° S) is characterized by an unexpectedly low diversity of arboreal and/or frugivorous mammals [14,50], potentially reflecting a scarcity of fruit owing to pronounced Patagonian seasonality [14,51]. Thus, Santa Cruz may be missing hypocarnivorous or mesocarnivorous species that would be present at a lower-latitude site of similar age. In this respect, it is worth noting that the two Neogene sparassodonts universally regarded as omnivorous, *Hondadelphys* and *Stylocynus* [11,20,52], come from extra-Patagonian localities [5]. Similarly, low-latitude species of *Lycopsis* (*L. longirostrus* and *L. padillai*) have proportionally larger molar grinding areas than Patagonian *L. torresi* (R. Engleman 2017, personal observation), suggesting more omnivorous habits. Additional sampling of middle- and low-latitude sites may eventually make it possible to test for regional differences in guild structure in South America.

Another possibility is that mesocarnivore and hypocarnivore niches at Santa Cruz were filled by mammals other than sparassodonts. The palaeothentid metatherian *Acdestis* may have included some vertebrate prey in its diet [53], perhaps warranting consideration as a small (approx. 350 g [54]) hypocarnivore. Other possible hypocarnivores include armadillos, particularly euphractines, which are more omnivorous than other extant armadillos and are known to catch and eat vertebrates [55]. Euphractines were the predominant armadillos during much of the Cenozoic [56], and some were apparently specialized for preying on vertebrates [57]. Armadillos may even have prevented sparassodonts from entering more omnivorous niches owing to ecological incumbency [58]. However, only a single Santa Cruz armadillo, *Prozaedyus*, may have had omnivorous habits like those of modern euphractines [59]. All other Santa Cruz mammals were primarily insectivorous, frugivorous and/or herbivorous [14,54,60]. Didelphoids are not recorded at Santa Cruz or many other fossil sites outside equatorial latitudes prior to the Late Miocene, and those that are known are small (less than 500 g) and non-carnivorous [52,61]. In summary, one or two non-sparassodont mammals could potentially be considered broadly hypocarnivorous, but the Santa Cruz carnivore guild would still be anomalous in its proportion of hypercarnivores and lack of mesocarnivores.

A fourth possible explanation is that current palaeodietary reconstructions of sparassodonts are inaccurate, and that not all Santa Cruz species were hypercarnivorous. This hypothesis cannot presently be tested, though it may be possible in the future using stable isotopes, dental wear or another ‘taxon-independent’ method of dietary inference [62,63]. Caution is warranted when interpreting the palaeobiology of any extinct clade based on extant representatives of other groups, as there is no unequivocal way to ‘calibrate’ boundaries of ecological categories (e.g. dietary categories) along a morphological continuum (e.g. relative grinding area). This phenomenon is well illustrated by South American notoungulates; most notoungulates have long been interpreted as grazers or open-habitat feeders based on their very high-crowned (hypsodont) teeth (e.g. [64]), but recent studies using stable isotopes and dental wear have demonstrated that a simple relationship between hypsodonty and diet does not hold for this group [65,66], despite its use for many other clades. Studies of mandible shape in sparassodonts have interpreted some species as more omnivorous (mesocarnivorous or hypocarnivorous) than suggested by their dentition [20,67], and this could reflect inaccuracies in interpreting sparassodont dental ecomorphology based on metrics derived from modern carnivorans. Interestingly, Marshall's [10] qualitative analysis of sparassodonts envisaged much greater dietary breadth in the group, with three Santa Cruz genera classified as large carnivores (*Acrocyon*, *Arctodictis*, *Borhyaena*), two as large omnivores (*Lycopsis*, *Prothylacynus*), and the remainder as small to medium carnivores or omnivores similar to modern mustelids, mephitids, canids and didelphids (see his fig. 1). Marshall [10] had little objective basis for this classification, but the resulting carnivore guild has a trophic structure similar to those of other continents (figure 4).

If most sparassodonts truly were hypercarnivorous, certain trophic niches in South America were apparently unoccupied by mammals during much of the Cenozoic. Sparassodonts appear to have become hypercarnivorous early in their evolutionary history [5], and this may have precluded them from later exploiting meso- and hypocarnivorous niches despite ecological opportunity. Such a scenario is compatible with the concept of a macroevolutionary ratchet that favours hypercarnivory and selects against omnivory, as has been described for many other groups of carnivorous mammals [68–70].

Our palaeoecological analyses suggest that carnivore guilds of ancient South America were not analogous to modern carnivore guilds or fossil guilds from other continents. This seems to be partly or principally owing to the particular clade (sparassodonts) that dominated mammalian carnivore niches there for most of the Cenozoic. Although no analyses of fossil carnivore guilds on other continents that lack carnivoramorphans have yet been published, placental ‘creodonts’ apparently displayed significant dietary breadth in Africa [71], and the same may also have been true of marsupials in Australia [72]. How sparassodonts were able to coexist with one another despite low trophic diversity is unclear. There is no strong evidence for character displacement in body size [23], and although some species clearly differed in postcranial morphology, locomotor habits can only currently be assessed for about half of Santa Cruz species. More precise characterization of the terrestrial predator guild of Santa Cruz and other localities in South America will require more complete specimens and additional analytical techniques. Our analyses highlight that the Santa Cruz carnivore guild, and probably the entire mammal community, was structured very differently from modern mammal communities. This presents a challenge for accurately characterizing the palaeoecology of this and other such faunas but also an opportunity to document ecological configurations of mammalian communities that extend beyond those that exist today.

## Supplementary Material

Figure S1 and Tables S1-12

## Data accessibility

Supporting data necessary to replicate this study are available in the electronic supplementary material, tables S1–12, or in cited publications. All codings are based on published specimens or ones housed in publicly accessible collections.
