# Supplementary material for: Diversity and disparity of sparassodonts (Metatheria) reveal non-analogue nature of ancient South American mammalian carnivore guilds
Source: Proc Biol Sci. 2018 Jan 3;285(1870):20172012. doi: 10.1098/rspb.2017.2012 (PMC5784193; doi:10.1098/rspb.2017.2012)
Supplement: Figure S1 and Tables S1-12 [file rspb20172012supp1.pdf]

Figure S1. Graphs of morphospace occupation of the South American terrestrial carnivore guild during selected late Oligocene through early Pliocene intervals. See table S8 for absolute ages. Abbreviations: COH, Colhuehuapian; DES, Deseadan; HUY, Huayquerian; LAV, Laventan; Mon, Montehermosan; SAN, Santacrucian.

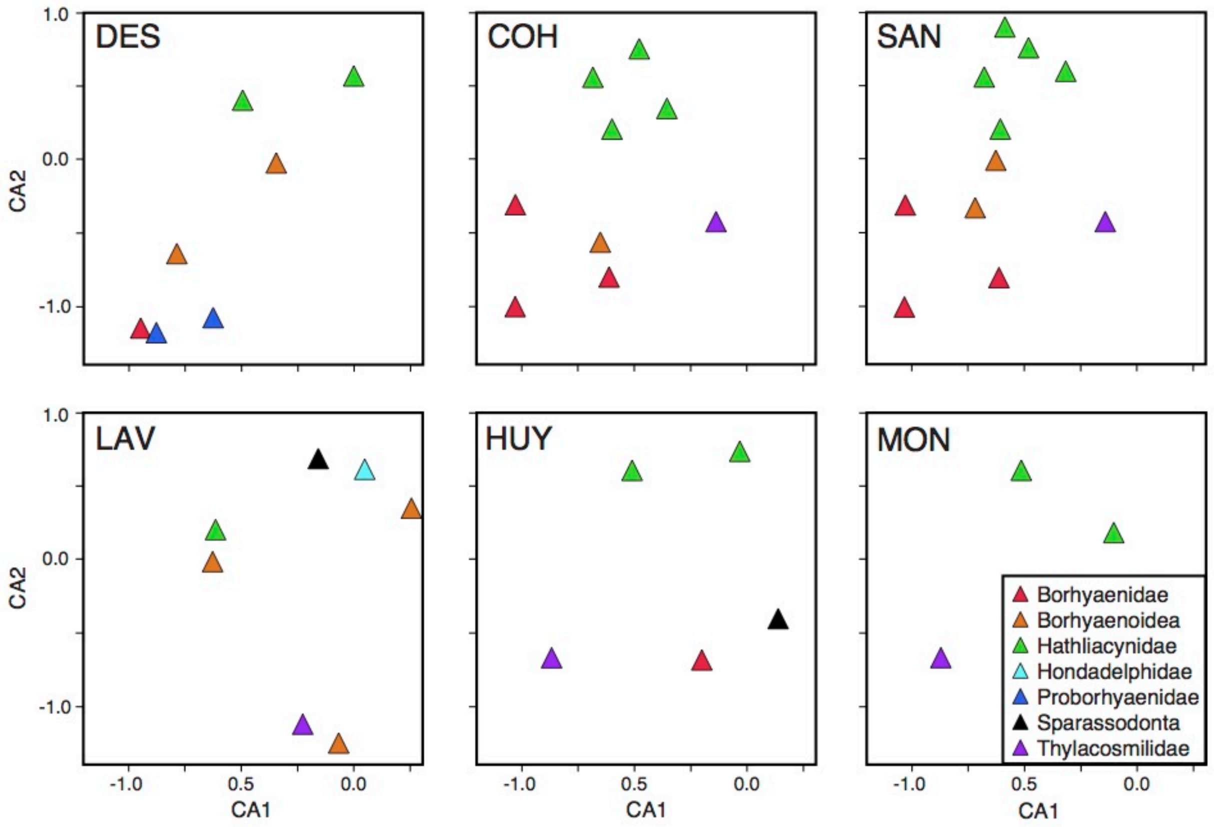

Table S1. List of carnivorous South American metatherian (Sparassodonta and Didelphimorphia) OTUs, higher taxa, estimated body masses, and temporal intervals in which they have been recorded. “Higher Taxon” refers to the least inclusive group to which the OTU can confidently be referred. Body mass ranges are based on previously published estimates and new estimates calculated for this study (tables S2 and S3, respectively), with preference given to estimates based on postcranial data. Temporal intervals (South American Land Mammal “Ages,” unless noted otherwise) are based on Forasiepi [1] and Zimicz [2], with modifications as described in the text and table S4. Post-Chapadmalalan occurrences of Didelphoidea (e.g., modern *Didelphis*) are not included. Abbreviations: Bar, Barrancan “Subage” of Casamayoran; Chp; Chapadmalalan; Chs, Chasicoan; Coc, Colloncuran (including Friasian sensu stricto); Coh, Colhuehuapian; Des, Deseadan; Huy, Huayquerian; Ita, Itaboraian; Lav, Laventan; LC, La Cantera locality (post-Tin, pre-Des); LF, Laguna Fría locality (post-Ita, pre-Vac); Mon, Montehermosan; Mus, Mustersan; Rio, Riochican; San, Santacrucian; Tin, Tinguiririrican; Vac, Vacan “Subage” of Casamayoran.

OTU	Higher Taxon	Body Mass (kg)	Temporal Interval(s)
Acrocyon	Borhyaenidae	21	Coh, San
Acyon	Hathliacynidae	6.8-13.2	Coh, San, Lav
Anachlysictis	Thylacosmilidae	48-83	Lav
Angelocabrerus	Borhyaenoidea	15.9-23.9	Bar
Arctodictis	Borhyaenidae	22.6-62	Coh, San, Coc
Arminiheringia	Proborhyaenidae	40.1	Bar

Australohyaena	Borhyaenidae	44-67	Des
Borhyaena	Borhyaenidae	16.4-36.4	Coh, San, Coc
Gen. et sp. indet.	Borhyaenidae	10.3-22.9	Huy
Borhyaenidium	Hathliacynidae	1.8-2.7	Huy, Mon, Chp?
Callistoe	Proborhyaenidae	19.9-25.7	Vac, Bar
Chasicostylus	Hathliacynidae	4.8-11.9	Chs
Cladosictis	Hathliacynidae	3.4-6.6	Coh, San, Coc
Didelphis	Didelphidae	0.4-0.6	Huy, Chp
Dukecynus	Borhyaenoidea	25-69	Lav
Fredszalaya	Borhyaenoidea	6.8-13.2	Des
Gen. et sp. nov. 6	Sparassodonta	1.1	LF
Hondadelphys	Sparassodonta	3.4-6.6	Lav
Hyperdidelphys	Didelphidae	1.0-2.1	Huy, Mon, Chp
IGM 251108	Borhyaenoidea	9.6-16.6	Lav
Lutreolina	Didelphidae	0.3	Huy, Mon, Chp
Lycopsis	Borhyaenoidea	17.1-29.8	San, Coc, Lav, Chs
MPEF-PV 4770	Hathliacynidae	3.7	Coh
Nemolestes	Sparassodonta	3.4-6.6	Ita, LF, Bar, Mus
Notictis	Hathliacynidae	0.7-1.1	Huy
Notocynus	Hathliacynidae	1.8-2.7	Mon
Notogale	Hathliacynidae	4.1	Des

Paraborhyaena	Proborhyaenidae	98-126	Des
Patagosmilus	Thylacosmilidae	25-60	Coh?, Coc
Patene	Sparassodonta	1.5-3.7	Ita, Rio, Bar, Tin
Perathereutes	Hathliacynidae	1.2	San
Pharsophorus	Borhyaenoidea	16.4-36.4	LC, Des
Plesiofelis	Borhyaenoidea	25-45	Mus
Proborhyaena	Proborhyaenidae	114-148	Des
Procladosictis	Sparassodonta	4.5-8.8	Mus
Prothylacynus	Borhyaenoidea	16.4-33	San, Coc
Pseudolycopsis	Borhyaenoidea	16.4-33	Chs
Pseudonotictis	Hathliacynidae	1.0-1.2	San, Coc
Pseudothylacynus	Borhyaenoidea	13.3-26.3	Coh
Sallacyon	Hathliacynidae	1.5	Des
Sipalocyon	Hathliacynidae	1.8-2.7	Coh, San, Coc
Sparassocynus	Sparassocynidae	0.4	Mon, Chp
Stylocynus	Sparassodonta	17.1-29.8	Huy
Thylacosmilus	Thylacosmilidae	48-117	Huy, Mon, Chp
Thylatheridium	Didelphidae	0.2-0.3	Huy, Chp
Thylophorops	Didelphidae	1.5-3.7	Huy, Chp
UF 27881	Sparassodonta	0.9	Lav

Table S2. Published mass estimates for sparassodonts and extinct didelphimorphians based on dental (d) and postcranial (p) measurements, rounded to the nearest 0.1 kg. Values in parentheses are minima and maxima.

**Taxon**	**Body Mass (Source)**
Acrocyon riggsi	17.0 kg [3](d)<br>26.3 kg [4] (d)
Acrocyon sectorius	11.5 kg [5] (d)<br>16.3 kg [3] (d)<br>28.7 kg [4] (d)
Acyon “herrerae”	7.0 kg [3] (d)<br>9.7 kg [4] (d)
Acyon myctoderos	12.0 kg [3] (d)
Acyon tricuspidatus	4.0 kg [4] (d)<br>4.3 kg [5] (d)<br>5.3 kg [3] (d)<br>8.0 kg [4] (d)[1]
Anachlysictis gracilis	16.1 kg [3] (d)<br>18.0 kg [4] (d)
Angelocabrerus daptes	17.1 kg [3] (d)
Arctodictis munizi	37 kg [6] (d)<br>43.0 kg [3] (d)<br>51.6 kg [4] (d)

Arctodictis sinclairi	18.3 kg [3] (d)<br>23.3 kg [4] (d)<br>39.9 kg (32.6-40.1 kg) [7] (p)
Arminiheringia auceta	31.3 kg [3] (d)
Arminiheringia contigua	24.0 kg [3] (d)
Arminiheringia cultrata	24.0 kg [3] (d)
Australohyaena antiqua	36.6 kg [3] (d)[2]<br>67.0 kg (44.1-67.1 kg) [8] (d)
Borhyaena macrodonta	31.3 kg [3] (d)<br>34.7 [4] (d)
Borhyaena tuberata	21.4 [4] (d)<br>23 kg (18.9-29.3 kg) [9] (p)<br>23.3 kg [6] (d)<br>28.5 kg (d)<br>36.4 kg (16.5-39.7 kg) [7] (p)
Borhyaenidium altiplanicus	1.2 kg [3] (d)
Borhyaenidium musteloides	1.6 kg [3] (d)
Borhyaenidium riggsi	2.0 kg [3] (d)
Callistoe vincei	19.9-25.7 kg [10] (p)<br>27.8 kg [3] (d)<br>31.0-34.3 kg [10] (p)
Chasicostylus castroi	6.7 kg [3] (d)

	9.8 kg [4] (d)
Cladosictis centralis	3.4 kg [3] (d)<br>4.4 kg [4] (d)
Cladosictis patagonica	3.4-8.7 kg [11] (p)<br>3.7 kg [6] (d)<br>4.0 kg [4] (d)<br>4.7 kg [3] (d)<br>6.6 kg (3.3-15.3 kg) [7] (p)
Didelphis crucialis	0.6 kg [2] (d)
Didelphis reigi	0.6 kg [2] (d)
Didelphis solimoensis	0.4 kg [2] (d)
Dukecynus magnus	24.6 kg [3] (d)<br>68.4 kg [4] (d)
Fredszalaya hunteri	20.6 kg [3] (d)
Hondadelphys fieldsi	3.7 kg [3] (d)
Hyperdidelphys dimartinoi	2.1 kg [2] (d)
Hyperdidelphys inexpectata	1.0 kg [2] (d)
Hyperdidelphys parvula	1.0 kg [2] (d)
Hyperdidelphys pattersoni	1.2 kg [2] (d)
Lutreolina tracheia	0.3 kg [2] (d)
Lycopsis longirostris	12.8 kg [4] (d)<br>17.1 kg (16.6-17.6 kg) [9] (p)

	29.8 kg (18.8-35.1 kg) [7] (p)<br>42.5 kg [3] (d)
"Lycopsis" torresi	19.4 kg [4] (d)<br>31.6 kg [3] (d)
"Lycopsis" viverensis	10.9 kg [3] (d)
Nemolestes spalacotherinus	4.95 kg [3] (d)
Notictis ortizi	0.9 kg [3] (d)
Notocynus hermosicus	1.8 kg [3] (d)<br>3.2 kg [4] (d)
Notogale mitis	2.7 kg [3] (d)
Paraborhyaena boliviana	24.0 kg [3] (d)
Patagosmilus goini	16.1 kg [3] (d)
Patene coluapiensis	2.5 kg [3] (d)
Perathereutes pungens	1.1 kg [3] (d)<br>2.5 kg [4] (d)
Pharsophorus lacerans	27.1 kg [3] (d)
Pharsophorus tenax	14.9 kg [3] (d)
Plesiofelis schlosseri	32.0 kg [3] (d)
Proborhyaena gigantea	153.6 kg [3] (d)
Procladosictis anomala	6.5 kg [3] (d)
Prothylacynus patagonicus	13.8 kg [6] (d)<br>20.6 kg [3] (d)

	26.8 kg [4] (d)<br>31.8 kg (23.2-45.4 kg) [7] (p)<br>33 kg (27.3-36.8 kg) [9] (p)
Pseudolycopsis cabrerai	14.4 kg [3] (d)<br>24.0 kg [4] (d)
Pseudonotictis chubutensis	0.9 kg [3] (d)
Pseudonotictis pusillus	0.9 kg [3] (d)<br>1.2 kg (1.0-1.2 kg) [7] (p)
Pseudothylacynus rectus	14.1 kg [4] (d)<br>19.8 kg [3] (d)
Sallacyon hoffstetteri	1.1 kg [3] (d)
Sipalocyon externa	0.9 kg [3] (d)
Sipalocyon gracilis	1.9 kg [6] (d)<br>2.0 kg [3] (d)<br>2.1 kg (1.9-4.0 kg) [7] (p)
Sipalocyon obtusa	1.8 kg [3] (d)<br>2.1 kg (1.9-4.0 kg) [7] (p)
Sparassocynus bahiai	0.4 kg [2] (d)
Sparassocynus derivatus	0.4 kg [2] (d)
Stylocynus paranensis	26.8 kg [3] (d)<br>35.3 kg [4] (d)
Thylacosmilus atrox	30.2 kg [3] (d)

	48-82 kg (37-107.9) [11] (p)<br><br>58.0 kg [4] (d)<br><br>116 kg [12] (p)<br><br>117.4 kg (56.1-137.9 kg) [7] (p)
Thylatheridium cristatum	0.2 kg [2] (d)
Thylatheridium hudsoni	0.2 kg [2] (d)
Thylatheridium pascuali	0.3 kg [2] (d)
Thylophorops perplanus	1.5 kg [2] (d)
Thylophorops chapadmalensis	3.7 kg [2] (d)

[1] Listed as *Anatherium defossus*

[2] Listed as *Pharsophorus ?antiquus*

Table S3: New sparassodont body mass estimates calculated for this study. Measurements are personal observations of DAC where no citation is provided. Abbreviations: cm, centimeter; EQ, regression equation; g, gram; HB, head-body; kg, kilogram; L, length; m, lower molar; M, upper molar; mm, millimeter; p, lower premolar.

**Species**	**Mass**	**Method and Other Notes**
Acrocyon riggsi	20.9 kg	Based on M1-4 L of 38 mm ([13]:fig. 12; EQ: [14]: dasyuromorphians).
Acyon myctoderos	6.8-13.2 kg	Based on resemblance to *Cladosictis* (below) and partial skeleton of *Acyon myctoderos* from Quebrada Honda currently under study (UATF-V-001400). Ls of femur, tibia, fibula, and calcaneum are approx. 25% longer than corresponding elements of large specimens of *Cladosictis* ([11]:appendix), suggesting a body mass roughly twice that of *Cladosictis*, assuming geometric similarity.
Anachlysictis gracilis	48-83 kg	Based on resemblance to smaller individuals of *Thylacosmilus* atrox (below). In *Anachlysictis*, mandible L is approximately 17.5 cm and m2-4 L is 42.7 mm ([15]:fig 11.6). The corresponding values for FMNH P 14333 are 17.5 cm ([16]:23) and 41.5 mm ([16]:fig 3).

Angelocabrerus daptes	15.9-23.9 kg	Based on m1, m2, and m3 L as reported by Simpson [17]:table 1 (EQ: [14]: dasyuromorphians).
Arctodictis munizi	62.0-68.6 kg	Based on resemblance to *A. sinclairi* (below). Lower molar row L of *A. munizi* averages 6.3 cm ([13]:table 10) versus 4.5 cm in *A. sinclairi* ([1]:table 4), suggesting a body mass 2.7x that of *A. sinclairi*, assuming geometric similarity.
Arctodictis sinclairi	22.6-25.0 kg	Based on HB L of 100 cm ([1]:96; EQs: [18]: light habitus; [19]: all mammals, carnivorans, marsupials).
Arminiheringia auceta	40.1 kg	Based on m1-4 L of 58.8 mm (MACN-A 10972; EQ: [14]: dasyuromorphians). The robust skull of *Arminiheringia* suggests it was more massive than closely-related *Callistoe* [20] even though m1-4 L is nearly identical (pers. observ.).
Borhyaena tuberata	16.4-18.2 kg	Based HB L of 90 cm ([9]:text-fig. 22; EQs: [18]: light habitus; [19]: all mammals, carnivorans, marsupials).

Borhyaeninae indet. (Entre Rios Fm.)	10.3-22.9 kg	Based on resemblance to *Borhyaena tuberata* (above). L of m2 of this specimen is 10.2 mm ([21]:figure 5), compared to an average of 11.9 mm in *Borhyaena tuberata* ([13]:table 6), suggesting a body mass nearly two-thirds that of *Borhyaena tuberata*, assuming geometric similarity.
Borhyaenidium musteloides	-	Same size as *Sipalocyon gracilis* (below). L of p1-m4 of MLP 57-X-10-153 is 41.8 mm, within the range of values for *Sipalocyon* (39.3-43.4 mm for MACN 5938 and MACN-A 691, respectively).
Borhyaenidium riggsi	-	Same size as *Sipalocyon gracilis* (below). L of p1-m4 of FMNH PM 14409 is 42.4 mm, within the range of values for *Sipalocyon* (39.3 mm and 43.4 mm for MACN 5938 and MACN-A 691, respectively).

Chasicostylus castroi	4.8-11.9 kg	Based on resemblance to *Cladosictis patagonica* (below). Ls of M1 and M2 of *Chasicostylus* measure 9.0 and 9.2 mm, respectively ([22]:29), versus an average of 7.4 mm and 8.1 mm, respectively in *Cladosictis* ([23]:table 20), suggesting a body mass 45-80% greater than that of *Cladosictis patagonica*, assuming geometric similarity.
Cladosictis patagonica	4.1-4.9 kg	Based on HB L of 57 cm ([24]:plate 61; EQs: [18]: light habitus; [19]: all mammals, carnivorans, marsupials).
Dukecynus magnus	24-42 kg	Based on resemblance to *Lycopsis longirostris* (below). L m2 of *Dukecynus* (15.5 mm; [15]:table 11.1) is about 10% longer than that of *L. longirostris* (13.8 mm; [25]:table 1), which suggests a body mass about 40% greater, assuming geometric similarity.
Fredszalaya hunteri	-	Based on *Acyon myctoderos* (above). L of calcaneum of *Fredszalaya* is 33.2 mm ([26]:appendix), comparable to the calcaneum of a partial skeleton of *Acyon myctoderos* from Quebrada Honda currently under study (UATF-V-001400).

Gen. et sp. nov. 6 (Paso del Sapo)	1.1 kg	Based on M3 L of 5.3 mm ([27]:fig. 3; EQ: [14]: dasyuromorphians).
Hondadelphys fieldsi	-	Based on resemblance to *Cladosictis* (above). The femur of *Hondadelphys* is about 10 cm long ([28]:414), within the range of *Cladosictis* (9.8-11 cm; [24]:390). The same is true of the breadth across its distal condyles (18.2 mm in *Hondadelphys* versus 17.5-21 mm in *Cladosictis*).
IGM 251108	9.6-16.6 kg	Based on resemblance to *Anachlysictis* (above). L of m2-4 of IGM 251108 is approx. 25 mm ([15]:fig. 11.7) compared to 42.7 mm in *Anachlysictis* (see above), suggesting a body mass about 20% that of *Anachlysictis*, assuming IGM 251108 is a geometrically similar thylacosmilid.
MPEF-PV 4770	3.7 kg	Based on m1-4 L of approx. 28 mm (EQ: [14]: dasyuromorphians).
Nemolestes spalacotherinus	-	Same size as *Cladosictis* (above; [29]:44; [13]:26).

Notictis ortizi	0.7-1.1 kg	Based on m1 L of 4.5 mm, m2 L of 4.8 m, and m1-4 L of 19 mm ([23]:table 2; EQ: [14]: dasyuromorphians).
Notocynus hermosicus	-	Same size as *Sipalocyon gracilis* (below). L of p1-m4 of MLP 11-91 (based on alveoli) is approx. 40 mm, within the range of values for *Sipalocyon* (39.3-43.4 mm for MACN 5938 and MACN-A 691, respectively).
Notogale mitis	4.1 kg	Based on m1-4 L of approx. 29 mm ([30]:table 1; EQ: [14]: dasyuromorphians).
Paraborhyaena boliviana	98-126 kg	Based on resemblance to *Callistoe* (above). L of m1-4 is 88 mm in *Paraborhyaena* (UATF-V-000129) compared to 51.7 mm in *Callistoe* ([20]:fig. 3), suggesting a body mass nearly five times that of *Callistoe*, assuming geometric similarity.
Patagosmilus goini	25-60 kg	Based on resemblance to *Thylacosmilus* (below). L of M1-3 is approximately 40 mm in *Patagosmilus* ([31]:fig 3) compared to 50 mm in *Thylacosmilus* ([16]:plate II), suggesting a body mass about 50% that of *Thylacosmilus*, assuming geometric similarity.

Patene coluapiensis	3.7 kg	Based on M1-4 L of 24.5 mm ([29]:44; EQ: [14] dasyuromorphians).
Patene simpsoni	1.5 kg	Based on M1-4 L of approx. 18.7 mm ([23]:table 1; EQ: [14]: dasyuromorphians).
Perathereutes pungens	1.2 kg	Based on m1-4 L of 19.7 mm (alveolar length of MACN-A 684; EQ: [14]: dasyuromorphians)
Pharsophorus lacerans	-	Same size as *Borhyaena* (above; [13]:32; [32]).
Plesiofelis schlosseri	25-45 kg	Based on resemblance to *Borhyaena* (above). L of m1-4 of *Plesiofelis* (58.8 mm; holotype, MLP 11-114) is more than 10% larger than that of *Borhyaena* (approx. 52 mm; [13]:table 6 and MACN-A 52-390), suggesting a body mass about 45% larger than that of *Borhyaena*, assuming geometric similarity. However, L of m1-4 of *Arminiheringia* is also 58.8 mm (holotype, MACN-A 10970), which suggests a slightly lower upper range.

Proborhyaena gigantea	114-148 kg	Based on resemblance to *Callistoe* (above). L of m1-4 is 92.5 mm in *Proborhyaena* (MACN 52-382) compared to 51.7 mm in *Callistoe* ([20]:fig. 3), suggesting a body mass nearly six times that of *Callistoe*, assuming geometric similarity.
Procladosictis anomala	4.5-8.8 kg	Based on resemblance to *Cladosictis* (above). M1, M2, and M3 L of *Procladosictis anomala* (9.0 mm, 9.5 mm, 7.5 mm, respectively; [23]:17) are approx. 10% larger than values for *Cladosictis* (mean: 7.4 mm, 8.1 mm, and 8.7 mm, respectively; [23]:table 20), suggesting a body mass about one-third greater than that of *Cladosictis*, assuming geometric similarity.
Prothylacynus patagonicus	16.4-18.2 kg	Based on HB L of 90 cm ([24]:plate 61; EQs: [18]: light habitus; [19]: all mammals, carnivorans, marsupials).
Pseudolycopsis cabrerai	-	Same size as *Prothylacynus patagonicus* ([33]:291-292).
Pseudonotictis pusillis	1.0 kg	Based on m1-4 L of 18.5 mm (MLP 11-26; EQ: [14]: dasyuromorphians).

Pseudothylacynus rectus	13.3-26.3 kg	Based on resemblance to *Prothylacynus patagonicus* (above). L of p1-m4 is 66.4 mm in *Pseudothylacynus* (MACN-A 52-369) compared to 71.6 mm in *Prothylacynus* (MACN 706), suggesting a body mass about 80% that of *Prothylacynus*, assuming geometric similarity.
Sallacyon hoffstetteri	1.5 kg	Based on m1-4 L of 21 mm ([34]:table 1; EQ: [14]: dasyuromorphians).
Sipalocyon gracilis	1.8-2.7 kg	Based on m1-4 L of 22.2 mm and 25.3 mm (MACN 5938 and MACN-A 691, respectively; EQ: [14]: dasyuromorphians).
Stylocynus paranensis	-	Same size as *Lycopsis longirostris*. Range of p1-m4 L in *Stylocynus* (95-115 mm based on alveolar L of MLP 11-94 and MLP 959, respectively) includes that of *Lycopsis* (100.5 mm; [25]:table 1).
UF 27881	900 g	Based on M1-4 alveolar L of 16.2 mm (EQ: [14]: dasyuromorphians).

Table S4. Additional information about OTUs included in this study (see also table S1) and excluded from this study.

**Included OTUs**	**Comments**
Acyon	Includes *Anatherium herrerae* [35].
Arminiheringia	Mustersan occurrence of this taxon was reinterpreted by Powell *et al.* [36] as a Barrancan occurrence of *Callistoe*.
Borhyaenidae indet.	Includes cf. *Borhyaena* from Entre Rios [21].
Cladosictis	Includes *Anatherium defossus* [35].
Gen. et sp. nov. 6	From Tejedor *et al.* [27]. Considered Sparassodonta incertae sedis rather than Hathliacynidae based on Forasiepi [1]. Recorded for the early Eocene “Laguna Fria interval” based on specimens from Laguna Fria; not recorded for other intervals due to uncertainties surrounding the age of La Barda (from which a referred partial tooth was collected).
IGM 251108	From Goin [15].
Lycopsis	Colloncuran occurrence based on Suarez *et al.* [37].
MPEF-PV 4770	From Goin *et al.* [38].
Patagosmilus	Described by Forasiepi and Carlini [31]. Assumed to include an undescribed Colhuehuapian specimen described as resembling an uncatalogued Colloncuran thylacosmilid [38]:58.

Patene	Tinguiririican occurrence based on Goin and Candela [39].
UF 27881	Described by Engelman and Croft [40].
**Excluded OTUs**	**Comments**
Allqokirus	From early Palaeocene of Tiupampa, Bolivia, which predates our comparative data set.
Argyrolestes	Holotype is lost and of unknown provenance [1].
Eutemnodus	Too fragmentary to code [21].
Gen. et sp. indet. C	From Paso del Sapo [27]. Status unknown, tooth position uncertain, no photo or metric data available.
Hathliacynidae?	From La Cancha [41]. Tooth position uncertain, not enough morphology preserved to be coded.
Mayulestes	From early Palaeocene of Tiupampa, Bolivia, which predates comparative data set, and sparassodont affinities uncertain [1,40].
New genus and species	From Pampa Grande of Lumbrera Formation [36]. Undescribed specimen not yet available for study.
Pseudocladosictis	Considered nomen vanum by [1].
PVL 4651	Probably a distinct taxon closely related to *Stylocynus* [42]. Not possible to code due to fragmentary nature and many ambiguous features (e.g., presence or absence of metaconid) due to wear.

Table S5. List of specimens studied and references consulted for coding sparassodont, dasyuromorphian, and didelphimorphian OTUs in this study. Abbreviations: ACM, Beneski Museum of Natural History, Amherst College, U.S.A.; AMNH, American Museum of Natural History, New York City, USA; CMNH, Cleveland Museum of Natural History, Cleveland, USA; FMNH, The Field Museum, Chicago, USA; MACN, Museo Argentino de Ciencias Naturales “Bernardino Rivadavia,” Buenos Aires, Argentina; MACN-A, Ameghino Collection, Museo Argentino de Ciencias Naturales “Bernardino Rivadavia,” Buenos Aires, Argentina; MLP, Museo de La Plata, La Plata, Argentina; MMH, Museo de Monte Hermoso, Monte Hermoso, Argentina; MMP, Museo Municipal de Ciencias Naturales "Lorenzo Scaglia", Mar del Plata, Argentina; MNHN-Bol, Museo Nacional de Historia Natural, La Paz, Bolivia; UATF, Universidad Autónoma Tomás Frías, Potosí, Bolivia; UCMP, University of California Museum of Paleontology, Berkeley, USA.

**Taxon**	**Specimen(s)**	**Reference(s)**
SPARASSODONTA		
Acrocyon riggsi	FMNH P13433	[38]
Acrocyon sectorius	MACN 9364-85 (cast)	-
Acyon “herrerae”	FMNH P 13521 (cast)	[35]
Acyon myctoderos	MNHN-Bol-V-003668 (cast of holotype)	[1,35]
Anachlysictis gracilis	-	[15]
Angelocabrerus daptes	-	[17]

Arctodictis munizi	MLP 11-85 (holotype)	[1,13]
Arctodictis sinclairi	MLP 85-VII-3-1	[1]
Arminiheringia auceta	MACN 10970 (holotype), MACN 10972	[20]
Australohyaena antiqua	-	[8]
Borhyaena macrodonta	MACN-A 52-366, MACN 52-390	[13]
Borhyaena tuberata	MACN-A 5780; MACN-A 6203-6265	[1,24]
Borhyaenidium musteloides	MLP 57-X-10-153 (holotype)	[23]
Borhyaenidium riggsi	FMNH P14409	[23]
Callistoe vincei	-	[10,20]
Chasicostylus castroi	MMH 84-4-7	[22]
Cladosictis patagonica	MACN-A 674, MACN-A 5927, MACN-A 5950	[1,23]
Dukecynus magnus	-	[15]
Fredszalaya hunteri	-	[26]
Gen. et sp. nov. 6	-	[27]
Hondadelphys fieldsi	UCMP 37960	[1,15,28,33]
IGM 251108	-	[15]

Lycopsis longirostrus	UCMP 38061	[1,15,25]
Lycopsis torresi	MLP 11-113 (holotype)	[43]
Lycopsis viverensis	MMH 87-6-1 (cast)	-
MPEF-PV 4770	MPEF-PV 4770	[38]
Nemolestes spalacotherinus	MACN-A 10330	[27]
Nemolestes sp.	-	[44]
Notictis ortizi	MACN-A 3996 (cast of holotype)	[23]
Notocynus hermosicus	MLP 11-91	[23]
Notogale mitis	ACM 3117 (cast), YPM-VPPU 21871	[23,32]
Paraborhyaena boliviana	UATF-V-000129	[34,45]
Patagosmilus goini	-	[31]
Patene simpsoni	-	[23,46]
Patene coluapiensis	-	[29]
Perathereutes pungens	MACN-A 684 (cast)	[23]
Pharsophorus lacerans	MACN-A 52-391	[13,32,34]
Plesiofelis schlosseri	MLP 11-114	-
Proborhyaena gigantea	AMNH 25976, MACN 52-382, MLP 79-XII-18-1	-

Procladosictis anomala	MACN-A 10327	-
Prothylacynus patagonicus	MACN-A 706-707, MACN-A 5269, MACN-A 5931, MACN 14453	[1,43]
Pseudolycopsis cabrerai	-	[33,43]
Pseudonotictis chubutensis	-	[47]
Pseudonotictis pusillus	MACN-A 666 (holotype), MLP 11-26	[23]
Pseudothylacynus rectus	MACN-A 52-369	[38]
Sallacyon hoffstetteri	-	[30,34]
Sipalocyon externa	MACN-A 52-383	-
Sipalocyon gracilis	MACN-A 691-692, MACN-A 5938	[1,23]
Stylocynus paranensis	MACN 5893, MACN 13203, MLP 11-94 (holotype), MLP 41-XII-13-959, MLP 41-XII-131112	[1,43]
Thylacosmilus atrox	FMNH P14531 (holotype), FMNH P14344 (paratype), MLP 35-X-4-1	[1,16,48]
UF 27881	UF 27881	-
DASYUROMORPHIA		

Dasyurus maculatus	CMNH 18912	-
Sarcophilus harrisii	CMNH 18915	-
Thylacinus cynocephalus	CMNH 18916	[1]
DIDELPHIMORPHIA		
Didelphis crucialis	MACN 604 (cast), MMP 879-M (cast)	-
Hyperdidelphys inexpectata	MACN 1615 (cast)	[49]
Hyperdidelphys parvula	MACN 5920 (cast), MACN 17781 (cast)	[49]
Lutreolina sp.	FMNH P14487 (cast)	-
Sparassocynus bahiai	MLP 11-92 (cast)	[50]
Sparassocynus derivatus	MACN 17909 (cast)	[50]
Thylatheridium cristatum	MACN 6442 (cast), MACN 6443 (cast)	-
Thylophorops chapadmalensis	MMP 354-S (cast), MMP 1037-M (cast)	[51]

Table S6. Character descriptions and comments on sparassodont codings. Characters #1-16 are from Wesley-Hunt [52], as modified by Werdelin and Wesley-Hunt [53], with the numerator and denominator reversed in Character #16 to make its calculation congruent with the original concept of relative grinding area [54] as well as its current usage (e.g., [3]). Bins for Character #16 follow Werdelin and Wesley-Hunt [53], except that bin "2" of that study was divided in two in order to better differentiate between hypocarnivorous and mesocarnivorous taxa (based on extant species with known diet). Character #17 (body mass) is based on Wesley-Hunt [52], with the smallest body size state divided into two. All multistate characters are ordered except #7 and #13. See Wesley-Hunt [52]:appendix for justification of original characters and codings. For characters #7-12, M3 and m4 were considered the carnassial teeth in sparassodonts, since these are the largest and least-worn slicing teeth in adult individuals. For characters #13-15, M4 was considered the sole post-carnassial tooth.

#	**Description**
1	Upper incisor row shape: (1) parabolic; (2) straight.
2	Cross-sectional shape of upper canine (mesiodistal/buccolingual diameter): (1) $X \leq 1.2$; (2) $1.2 < X \leq 1.35$; (3) $1.35 < X \leq 1.5$; (4) $1.5 < X \leq 1.7$; (5) $X > 1.7$. Measured at the alveolar border.
3	Number of upper premolars anterior to carnassial: (0) none; (1) 1; (2) 2; (3) 3.
4	Shape of largest upper premolar anterior to carnassial in occlusal view (length/width): (1) $X < 1.7$; (2) $1.7 < X \leq 2.3$; (3) $X > 2.3$. Measured at base of crown.

5	Diastemata among upper premolars: (1) small or absent; (2) present.
6	Proportions of last lower premolar in occlusal view (length/width): (1) $X < 1.7$; (2) $1.7 \leq X \geq 2.2$; (3) $X > 2.2$. Measured at base of crown.
7	Upper carnassial shape in occlusal view: (1) rectangular; (2) triangular and equilateral; (3) triangular and elongate (approximating a right scalene triangle); (4) linear (very reduced protocone).
8	Upper carnassial relative blade length: (1) blade absent; (2) < 0.33; (3) 0.33-0.67; (4) 0.68-0.99; (5) entire tooth acts as blade. Blade length was measured along the lingually positioned postmetacrista. Total tooth length was measured parallel to this, from the protocone to the distal end of the stylar shelf.
9	Lower carnassial relative blade length: (1) 0; (2) $0 < x < 0.55$; (3); 0.55-0.75; (4) 0.75-0.9; (5) > 0.9. Blade length was measured along the buccal face, from paraconid to protoconid. Total tooth length was measured parallel to this, from paraconid to distal end of the talonid.
10	Angle of upper carnassial cusps in occlusal view: (1) $X < 24°$; (2) $25° \leq X < 30°$; (3) $30° \leq X < 40°$; (4) $X \geq 40°$. Measured as the angle between the protocone and paracone using the distal end of the stylar shelf as the vertex.
11	Angle of lower carnassial cusps in buccal view: (1) $0 < X <15°$; (2) $15 \leq X < 30°$; (3) $30° \leq X < 50°$; (4) $50° \leq X < 70°$; (5) $X \geq 70°$. Measured as angle at which line connecting tip of protoconid and highest point on the talonid intersects the horizontal plane.

12	Angle of lower carnassial trigonid cusps in occlusal view: (1) X = 0°; (2) 0° < X ≤ 40°; (3) 40° < X ≤ 80°; (4) 80° < X ≤ 130°; (5) X = 180°. Measured as angle between paracristid and metacristid (vertex at protoconid). Most sparassodonts lack a metaconid on m4 and were coded as 4.
13	Shape of upper first post-carnassial tooth: (1) square to elongate rectangular (longer than wide); (2) wide rectangular (wider than long); (3) triangular; (4) small or absent.
14	Number of postcarnassial upper teeth: (0) none; (1) 1; (2) 2; (3) 3.
15	Cusp shape of first post-carnassial upper tooth: (1) rounded; (2) sharp.
16	Relative grinding area of lower molars: (1) X = 1; (2) 1 > X ≥ 0.6; (3) 0.6 > X ≥ 0.44; (4) 0.44 > X ≥ 0.25; (5) 0.25 > X ≥ 0.167; (6) X < 0.167. Measured as talonid area of lower molars divided by total occlusal area of lower molars.[1]
17	Body mass: (1) x < 1.5 kg; (2) 1.5 ≤ x < 7 kg; (3) 7 ≤ x < 21.5 kg; (4) 21.5 ≤ x < 50 kg; (5) 50 ≤ x ≤ 100 kg; (6) > 100 kg.

[1] Relative grinding area (RGA) in sparassodonts has traditionally been calculated based solely on the last lower molar (m4). We calculate this value based on the entire tooth row for several reasons. First, although m4 may be the most specialized shearing tooth in sparassodonts [3,55], all lower molars function together as a single shearing complex to some degree. This also occurs in modern carnivorous dasyuromorphians, where RGA has been based on m3-4 [56]. Second, the morphology of m4 is not representative of the other lower molars in sparassodonts; m1–3 often have distinct talonids, whereas

the talonid of m4 is greatly reduced. Although a reduced m4 talonid characterizes many metatherians [57-61], this is generally expressed to a much greater degree in sparassodonts. For example, in the hathliacynid *Cladosictis patagonica*, the talonid of m3 is more than twice the size of that of m4; in some borhyaenoids, a basined talonid is present in m1–3 but absent on m4. Therefore, measuring RGA in sparassodonts using only m4 likely underestimates the total grinding area of the tooth row in many cases. Finally, this method of calculating RGA parallels that often used for carnivorans [54], facilitating direct comparisons with carnivorous placentals.

Table S7. Character-state codings for sparassodonts, didelphimorphians, and dasyuromorphians. See table S6 for character descriptions. Blue cells indicate states coded based on lower teeth or serial homologs. Red cells indicate states coded based on a closely-related and morphologically similar taxon (*Cladosictis* for *Chasicostylus*, *Perathereutes* for *Sallacyon*, and *Proborhyaena* for *Paraborhyaena*).

**Taxon**	**1**	**2**	**3**	**4**	**5**	**6**	**7**	**8**	**9**	**10**	**11**	**12**	**13**	**14**	**15**	**16**	**17**
Borhyaenidae_*Acrocyon*	?	3	3	1	1	1	4	4	5	1	5	6	3	1	2	6	3
Borhyaenidae_*Arctodictis*	2	2	3	1	1	2	4	4	5	1	5	6	3	1	2	6	5
Borhyaenidae_*Australohyaena*	2	2	3	1	1	1	4	4	4	1	4	6	3	1	2	6	5
Borhyaenidae_*Borhyaena*	2	3	3	1	1	2	4	4	4	2	4	6	3	1	2	6	4
Borhyaenidae_indet	?	?	?	?	?	?	?	?	4	?	4	6	?	?	?	?	4
Borhyaenoidea_*Angelocabrerus*	?	?	?	?	?	1	?	?	5	?	5	6	?	?	?	6	4
Borhyaenoidea_*Dukecynus*	?	1	3	?	1	?	3	?	?	?	?	?	?	?	2	?	5
Borhyaenoidea_*Fredszalaya*	?	?	3	1	1	?	3	3	?	1	?	?	3	1	2	?	3
Borhyaenoidea_*Lycopsis*	?	2	3	3	2	3	3	3	3	1	4	6	3	1	2	5	4
Borhyaenoidea_IGM251108	?	4	3	?	2	3	?	?	3	?	?	?	?	?	?	?	3
Borhyaenoidea_*Pharsophorus*	2	3	3	1	1	2	3	?	4	1	4	4	3	1	2	6	4
Borhyaenoidea_*Plesiofelis*	?	?	?	?	?	2	?	?	4	?	4	4	?	?	?	6	4
Borhyaenoidea_*Prothylacynus*	2	3	3	2	2	2	3	4	4	1	4	6	3	1	2	6	4
Borhyaenoidea_*Pseudolycopsis*	?	?	3	3	2	?	3	4	?	2	?	?	?	?	2	?	4
Borhyaenoidea_*Pseudothylacynus*	?	?	?	?	1	2	3	3	3	1	4	6	?	?	?	5	4
Hathliacynidae_*Acyon*	2	3	3	2	2	3	3	3	4	1	4	6	3	1	2	5	3
Hathliacynidae_*Borhyaenidium*	?	3	3	?	2	3	3	3	4	1	4	6	?	?	2	5	2
Hathliacynidae_*Chasicostylus*	?	?	3	?	?	?	4	4	4	1	4	6	?	?	2	5	3

Hathliacynidae_*Cladosictis*	2	3	3	1	2	3	4	4	4	1	4	6	3	1	2	5	2
Hathliacynidae_MPEFPV4770	?	?	3	?	2	3	?	?	4	?	4	6	?	1	?	5	2
Hathliacynidae_*Notictis*	?	?	?	?	?	?	?	?	?	?	?	?	?	?	?	?	1
Hathliacynidae_*Notocynus*	?	?	?	?	?	?	?	?	3	?	?	6	?	?	?	?	2
Hathliacynidae_*Notogale*	1	3	3	?	2	3	3	?	3	2	4	6	?	?	2	?	2
Hathliacynidae_*Perathereutes*	?	?	?	?	?	?	?	?	3	?	4	6	?	?	?	4	1
Hathliacynidae_*Pseudonotictis*	?	?	?	?	?	3	3	3	3	1	3	6	?	?	2	5	1
Hathliacynidae_*Sallacyon*	?	?	?	?	?	?	3	3	3	1	?	6	3	1	2	?	2
Hathliacynidae_*Sipalocyon*	2	3	3	2	2	3	3	3	3	1	4	6	3	1	2	4	2
Hondadelphidae_*Hondadelphys*	?	5	3	2	1	3	3	3	3	2	3	4	3	1	2	4	2
Proborhyaenidae_*Arminiheringia*	?	3	3	2	1	1	4	4	4	1	4	6	3	1	2	6	4
Proborhyaenidae_*Callistoe*	1	3	3	1	1	2	4	4	4	1	4	6	3	1	2	6	4
Proborhyaenidae_*Paraborhyaena*	1	3	3	?	1	?	?	?	5	1	5	6	3	1	2	6	6
Proborhyaenidae_*Proborhyaena*	?	5	3	?	1	1	?	4	5	1	5	6	?	?	?	6	6
Sparassodonta_Genetspnov6	?	?	?	?	?	?	3	?	?	?	?	?	?	?	2	?	1
Sparassodonta_*Nemolestes*	?	?	?	?	?	2	?	?	3	?	?	4	?	?	?	?	2
Sparassodonta_*Patene*	?	?	?	2	?	3	3	3	?	1	3	3	3	1	2	?	2
Sparassodonta_*Procladosictis*	?	?	?	2	?	?	3	4	?	1	?	?	?	?	?	?	3
Sparassodonta_*Stylocynus*	?	?	?	?	2	3	3	3	3	2	3	3	?	?	2	4	4
Sparassodonta_UF27881	?	2	3	?	1	?	?	?	?	?	?	?	?	1	?	?	1
Thylacosmilidae_*Anachlysictis*	?	?	?	?	1	?	?	?	4	?	4	6	?	?	?	?	5
Thylacosmilidae_*Patagosmilus*	?	5	2	2	2	?	3	4	?	1	?	?	3	1	2	?	5
Thylacosmilidae_*Thylacosmilus*	2	5	2	3	1	1	4	4	5	1	5	6	3	1	2	6	5
Dasyuridae_*Dasyurus*	1	2	2	1	2	2	3	3	3	1	4	3	3	1	2	4	2
Dasyuridae_*Sarcophilus*	1	1	2	1	1	1	4	4	4	1	4	5	3	1	2	5	3

Thylacinidae_*Thylacinus*	1	3	3	2	2	2	3	3	3	1	4	6	3	1	2	4	4
Didelphidae_*Didelphis*	1	5	3	1	2	1	3	3	2	1	3	3	3	1	2	4	1
Didelphidae_*Hyperdidelphys*[1]	1	4	3	1	1	1	3	3	3	1	4	3	3	1	2	4	1
Didelphidae_*Lutreolina*	1	5	3	1	2	3	3	3	3	1	3	3	3	1	2	4	1
Didelphidae_*Thylatheridium*	?	4	3	1	1	1	3	3	3	1	3	3	3	1	2	4	1
Didelphidae_*Thylophorops*	1	5	3	1	2	2	3	3	3	1	3	3	3	1	2	4	2
Sparassocynidae_*Sparassocynus*	2	3	3	1	1	1	3	3	3	1	4	3	3	1	2	4	1
Amphicyonidae_*Amphicyon*	1	3	3	2	2	2	4	4	3	1	3	3	2	2	1	2	6
Amphicyonidae_*Brachyrhynchocyon*	1	1	3	2	2	2	4	4	3	1	3	4	2	2	2	3	3
Amphicyonidae_*Daphoenodon*	1	2	3	2	2	2	4	4	3	2	3	4	2	2	1	2	5
Amphicyonidae_*Daphoenus*	1	3	3	2	2	3	4	3	3	3	3	3	2	2	2	2	3
Amphicyonidae_*Ischyrocyon*	1	2	3	1	2	2	4	4	3	2	3	6	2	2	1	2	6
Amphicyonidae_*Paradaphoenus*	2	4	3	2	2	3	4	3	3	1	3	4	2	2	2	2	2
Amphicyonidae_*Pliocyon*	1	3	3	2	1	2	4	4	3	2	2	4	2	2	1	2	6
Amphicyonidae_*Temnocyon*	1	4	3	2	2	3	3	4	3	3	3	4	2	2	2	3	4
Barbourofelidae_*Barbourofelis*	1	5	1	2	1	3	4	4	5	1	5	6	4	0	2	6	5
Canidae_*Aelurodon*	2	3	3	2	1	1	4	4	4	1	4	4	2	1	2	3	3
Canidae_*Borophagus*	2	?	3	2	1	1	4	3	3	1	3	4	2	1	1	3	3
Canidae_*Caedocyon*	2	3	3	2	1	?	4	4	?	2	1	?	2	1	2	?	3
Canidae_*Canis*_2	1	4	3	2	2	2	4	4	3	1	3	4	2	2	2	3	3
Canidae_*Carpocyon*	1	?	3	2	1	1	4	3	4	1	3	4	1	2	1	3	3
Canidae_*Cerdocyon*	1	4	3	3	1	3	4	4	3	1	3	4	2	2	2	2	3
Canidae_*Chrysocyon*	1	4	3	2	2	2	4	4	3	1	3	4	2	2	2	2	3
Canidae_*Cormocyon*	2	?	3	2	2	2	4	4	3	1	3	4	2	2	2	2	2
Canidae_*Cynarctoides*	?	?	3	2	1	2	4	4	3	1	2	4	2	2	2	2	2

Canidae_*Cynarctus*	1	?	3	2	2	2	4	3	2	1	2	4	1	2	2	2	3
Canidae_*Cynodesmus*	1	2	3	2	1	2	4	4	3	1	3	4	2	2	2	3	3
Canidae_*Ectopocynus*	?	?	?	?	?	1	4	4	3	1	4	6	2	1	2	3	3
Canidae_*Enhydrocyon*	2	3	2	1	1	1	4	4	3	1	4	6	2	1	2	4	3
Canidae_*Epicyon*	1	?	3	2	2	1	4	4	4	1	3	5	2	1	1	3	4
Canidae_*Euoplocyon*	1	?	3	2	1	2	4	4	3	1	3	6	2	1	2	4	3
Canidae_*Hesperocyon*	1	4	3	2	2	3	4	4	3	1	3	3	2	2	2	3	2
Canidae_*Leptocyon*	1	3	3	2	2	3	4	4	3	1	3	4	2	2	2	2	2
Canidae_*Mesocyon*	1	3	3	2	2	3	4	4	3	1	3	4	2	2	2	3	3
Canidae_*Osbornodon*_1	1	3	3	2	1	2	4	4	3	1	3	4	2	2	2	3	2
Canidae_*Osbornodon*_2	1	4	3	2	1	2	4	4	3	1	3	4	2	2	2	4	4
Canidae_*Otarocyon*	1	?	3	2	1	2	4	4	3	1	3	4	2	2	2	3	2
Canidae_*Paracynarctus*	1	?	3	2	1	2	4	3	3	2	3	4	1	2	2	2	3
Canidae_*Paraenhydrocyon*	1	3	3	2	2	2	4	4	3	1	3	4	2	2	2	3	3
Canidae_*Paratomarctus*	1	?	3	2	1	2	4	3	3	1	3	4	2	2	2	3	3
Canidae_*Philotrox*	1	1	2	2	1	2	4	4	3	2	3	4	2	1	2	3	3
Canidae_*Phlaocyon*	2	?	3	1	1	1	3	3	3	4	3	4	2	2	2	2	2
Canidae_*Psalidocyon*	1	?	3	2	1	2	4	4	3	1	3	4	2	2	2	3	3
Canidae_*Sunkahetanka*	1	2	3	1	1	1	4	4	3	2	3	4	2	1	2	3	3
Canidae_*Tephrocyon*	1	3	3	2	1	2	4	4	3	1	3	4	2	2	2	3	3
Canidae_*Tomarctus*	1	?	3	2	1	2	4	3	3	1	3	4	2	2	2	2	3
Canidae_*Urocyon*	1	4	3	2	2	2	4	4	3	1	3	4	2	2	2	3	2
Canidae_*Vulpes*	1	4	3	3	2	3	4	4	3	1	3	4	2	2	2	3	3
Felidae_*Felis*_6	2	2	1	2	1	2	4	4	5	1	5	6	4	0	2	6	4
Felidae_*Homotherium*	1	5	0	1	1	3	4	4	5	1	5	6	4	0	2	6	6

Felidae_*Lynx*	2	2	1	2	1	2	4	4	5	1	5	6	4	0	2	6	3
Felidae_*Machairodus*	1	5	1	3	1	3	4	4	5	1	5	6	4	0	2	6	6
Felidae_*Nimravides*	2	5	1	2	1	2	4	4	4	1	5	6	3	1	2	6	6
Felidae_*Pseudaelurus*	2	5	1	2	1	2	4	4	5	1	5	6	3	1	2	6	4
Felidae_*Smilodon*	1	5	1	2	1	3	4	4	5	1	5	6	4	0	2	6	6
Hyaenidae_*Chasmaporthetes*	?	2	3	1	1	2	4	4	4	1	5	6	4	0	2	6	6
Mephitidae_*Martinogale*	?	?	?	?	?	2	4	4	3	1	3	3	2	1	2	3	1
Mephitidae_*Mephitis*	1	2	2	1	1	1	3	4	3	3	2	3	1	1	2	3	2
Mephitidae_*Spilogale*	1	3	2	2	1	1	3	4	3	3	3	3	1	1	2	3	1
Miacoidea_*Bryanictis*	?	?	3	2	1	2	3	3	5	3	3	3	2	2	2	3	1
Miacoidea_*Didymictis*	2	3	3	2	1	3	4	3	2	2	4	3	2	2	2	3	3
Miacoidea_*Miacis*	?	3	3	1	1	2	4	4	3	2	4	3	2	2	2	2	2
Miacoidea_*Oodectes*	1	4	3	1	2	2	3	3	3	3	3	3	3	3	2	2	1
Miacoidea_*Palaeogale*	2	1	3	1	1	2	4	4	4	1	4	6	3	1	2	4	1
Miacoidea_*Procynodictis*	?	3	3	2	1	3	3	4	3	1	4	3	2	1	2	3	2
Miacoidea_*Tapocyon*	2	3	3	1	1	2	4	3	3	3	4	3	2	1	2	3	3
Miacoidea_*Uintacyon*	?	?	3	2	1	3	3	3	3	3	3	3	3	2	2	3	2
Miacoidea_*Vassacyon*	?	?	?	?	?	2	3	3	3	3	3	3	2	2	2	2	2
Miacoidea_*Viverravus*	1	3	3	2	2	2	4	4	3	1	4	3	2	2	2	3	2
Miacoidea_*Vulpavus*	1	3	3	1	1	2	3	2	3	3	2	3	2	2	2	2	1
Mustelidae_*Brachypsalis*	2	2	2	1	1	1	4	3	3	1	2	4	2	2	2	3	3
Mustelidae_*Craterogale*	?	?	2	1	1	?	3	4	3	3	2	3	1	1	2	4	2
Mustelidae_*Leptarctus*	2	1	2	1	1	?	1	3	3	4	2	3	1	1	2	3	2
Mustelidae_*Lutravus*	2	2	3	1	1	2	3	3	3	3	3	4	2	1	2	4	3
Mustelidae_*Martes*	2	2	3	2	1	2	4	4	3	2	3	4	2	1	2	3	1

Mustelidae_*Megalictis*	2	2	3	1	1	2	4	4	4	2	3	6	2	1	1	4	4
Mustelidae_*Miomustela*	2	3	2	2	1	?	4	4	3	1	3	4	2	1	2	4	1
Mustelidae_*Mionictis*	2	2	2	1	1	2	3	4	3	3	2	4	1	1	2	2	3
Mustelidae_*Mustela*	2	2	2	2	1	2	4	4	3	3	2	6	2	1	2	4	1
Mustelidae_*Oligobunis*	2	2	3	1	1	2	4	4	3	2	3	4	2	1	2	4	3
Mustelidae_*Plesiogulo*	2	2	3	1	1	1	4	3	3	2	3	4	2	1	1	4	5
Mustelidae_*Plionictis*	2	3	3	3	1	?	4	4	3	2	3	4	2	1	2	4	2
Mustelidae_*Pliotaxidea*	2	2	2	1	1	2	2	3	2	4	1	3	1	1	2	3	2
Mustelidae_*Potamotherium*	?	1	2	1	1	2	3	4	3	4	3	4	2	1	2	3	2
Mustelidae_*Promartes*	2	3	3	2	1	2	4	3	3	2	3	3	2	1	2	3	2
Mustelidae_*Sthenictis*	2	3	3	2	1	3	4	4	3	2	3	4	2	1	2	3	3
Mustelidae_*Taxidea*	2	2	2	1	1	2	1	3	3	4	3	4	1	1	2	3	3
Mustelidae_*Zodiolestes*	2	4	3	2	1	2	4	3	3	2	3	4	2	1	2	4	2
Nimravidae_*Dinictis*	2	5	2	2	2	3	4	4	4	1	4	6	3	1	2	5	4
Nimravidae_*Hoplophoneus*	1	5	2	2	1	3	4	4	4	1	5	6	3	1	2	6	5
Nimravidae_*Nimravus*	2	5	1	2	1	3	4	4	4	1	5	6	4	0	2	6	6
Nimravidae_*Pogonodon*	2	4	1	2	1	1	4	4	4	1	4	6	3	1	2	6	5
Procyonidae_*Bassariscus*	2	3	3	2	2	2	3	3	3	3	3	3	2	2	2	3	1
Procyonidae_*Edaphocyon*	2	4	3	1	2	1	1	1	2	4	1	3	1	2	2	2	3
Procyonidae_*Nasua*	1	5	3	1	2	1	2	1	2	4	2	3	1	2	2	1	2
Procyonidae_*Procyon*	1	3	3	1	1	1	1	1	3	4	1	3	1	2	2	1	2
Ursidae_*Agriotherium*	?	3	3	2	1	2	2	3	3	4	2	6	1	2	1	2	6
Ursidae_*Arctodus*	1	3	0	1	1	1	1	4	3	4	2	5	1	2	1	2	6
Ursidae_*Cephalogale*	2	2	3	2	1	1	3	4	3	3	3	4	2	2	1	3	5
Ursidae_*Hemicyon*	1	4	3	3	1	2	3	4	3	3	3	5	1	2	1	2	5

Ursidae_*Ursavus*	?	?	3	2	1	?	2	3	3	4	2	3	1	2	2	2	5
Ursidae_*Ursus*	2	3	0	?	1	?	1	1	3	4	2	2	1	2	1	1	5
Ailuridae_*Ailurus_fulgens*	1	4	2	1	1	1	1	1	3	3	1	3	1	2	2	1	2
Canidae_*Alopex_lagopus*	1	4	3	2	1	3	4	4	3	1	3	4	2	1	2	3	2
Canidae_*Atelocynus_microtis*	1	3	3	3	2	2	4	4	3	1	2	4	2	2	2	3	3
Canidae_*Canis_adustus*	1	5	3	3	2	3	4	4	3	1	3	4	2	2	2	2	3
Canidae_*Canis_aureus*	1	4	3	3	2	2	4	4	3	1	3	4	2	2	2	3	3
Canidae_*Canis_latrans*	1	4	3	3	2	3	4	4	3	1	3	4	2	2	2	3	3
Canidae_*Canis_lupus*	1	4	3	2	2	2	4	4	3	1	3	4	2	2	2	3	4
Canidae_*Canis_mesomelas*	1	4	3	3	2	3	4	4	3	1	3	4	2	2	2	3	3
Canidae_*Canis_rufus*	1	5	3	3	2	2	4	4	3	1	3	4	2	2	2	3	4
Canidae_*Canis_simensis*	1	4	3	3	2	2	4	4	3	1	3	4	2	2	2	3	3
Canidae_*Cerdocyon_thous*	1	4	3	3	2	3	4	4	3	1	3	4	2	2	2	3	2
Canidae_*Chrysocyon_brachyurus*	1	4	3	2	2	2	4	4	3	1	3	4	2	2	2	2	4
Canidae_*Cuon_alpinus*	1	4	3	3	1	2	4	4	3	1	3	4	3	1	2	4	3
Canidae_*Lycaon_pictus*	1	3	3	2	1	2	4	4	3	1	3	4	2	1	2	3	4
Canidae_*Nyctereutes_procyonoides*	1	4	3	3	1	2	4	4	3	1	3	4	1	2	2	3	2
Canidae_*Otocyon_megalotis*	1	2	3	2	2	2	3	3	2	3	2	3	2	3	2	2	2
Canidae_*Pseudalopex_culpaeus*	1	4	3	3	2	3	4	4	3	1	3	4	2	2	2	3	3
Canidae_*Pseudalopex_griseus*	1	4	3	3	2	3	4	4	3	1	3	4	2	2	2	2	3
Canidae_*Pseudalopex_gymnocercus*	1	4	3	3	2	3	4	4	3	1	3	4	2	2	2	3	2
Canidae_*Pseudalopex_sechurae*	1	4	3	3	2	3	4	4	3	1	3	4	2	2	2	2	2
Canidae_*Pseudalopex_vetulus*	1	4	3	3	2	2	4	4	3	1	3	4	2	2	2	2	2
Canidae_*Speothos_venaticus*	1	2	3	1	1	2	4	4	3	1	3	5	2	1	2	4	2
Canidae_*Urocyon_cinereoargenteus*	1	4	3	2	2	2	4	4	3	1	3	4	2	2	2	3	2

Canidae_*Urocyon_littoralis*	1	3	3	3	2	3	4	4	3	1	3	4	2	2	2	2	2
Canidae_*Vulpes_bengalensis*	1	4	3	3	2	3	4	4	3	1	3	4	2	2	2	2	2
Canidae_*Vulpes_chama*	1	5	3	3	2	3	4	4	3	1	3	4	2	2	2	2	2
Canidae_*Vulpes_rueppelli*	1	3	3	3	2	3	4	4	3	1	3	4	2	2	2	2	2
Canidae_*Vulpes_velox*	1	4	3	3	2	3	4	4	3	1	3	4	2	2	2	3	2
Canidae_*Vulpes_vulpes*	1	4	3	3	2	3	4	4	3	1	3	4	2	2	2	3	2
Canidae_*Vulpes_zerda*	1	3	3	3	2	3	4	4	3	1	3	4	2	2	2	2	1
Canidae_*Vulpes_corsac*	1	4	3	3	2	3	4	4	3	1	3	4	2	2	2	3	2
Canidae_*Vulpes_pallida*	1	4	3	3	2	3	4	4	3	1	3	4	2	2	2	2	2
Eupleridae_*Cryptoprocta_ferox*	2	2	1	1	1	2	4	4	4	1	4	5	4	0	2	6	3
Eupleridae_*Eupleres_goudotii*	1	4	3	3	2	3	4	3	2	2	1	3	3	2	2	3	2
Eupleridae_*Fossa_fossana*	1	2	3	2	2	2	4	3	3	2	3	3	2	2	2	2	2
Eupleridae_*Galidia_elegans*	2	4	2	2	1	2	4	4	3	1	4	3	3	1	2	4	1
Eupleridae_*Galidictis_fasciata*	2	3	2	1	1	1	3	4	3	2	3	3	3	2	2	3	1
Eupleridae_*Galidictis_grandidieri*	2	2	2	1	1	1	3	3	3	2	4	3	3	2	2	3	2
Eupleridae_*Salanoia_concolor*	2	3	2	1	1	2	3	3	3	2	2	3	3	2	2	3	1
Felidae_*Acinonyx_jubatus*	2	2	1	3	1	3	4	5	5	1	5	5	4	0	2	6	5
Felidae_*Caracal_caracal*	2	3	1	2	1	2	4	4	5	1	5	5	4	0	2	6	3
Felidae_*Catopuma_badia*	2	2	1	2	1	2	4	4	5	1	5	5	4	0	2	6	2
Felidae_*Catopuma_temminckii*	2	2	1	2	1	2	4	4	5	1	5	5	4	0	2	6	3
Felidae_*Felis_bieti*	2	2	1	2	1	2	4	4	5	1	5	5	4	0	2	6	2
Felidae_*Felis_chaus*	2	2	1	2	1	3	4	4	5	1	5	5	4	0	2	6	3
Felidae_*Felis_margarita*	2	3	1	2	1	3	4	4	5	1	5	5	4	0	2	6	2
Felidae_*Felis_nigripes*	2	3	1	3	1	3	4	5	5	1	5	5	4	0	2	6	2
Felidae_*Felis_silvestris*	2	3	1	2	1	2	4	4	5	1	5	5	4	0	2	6	2

Felidae_*Herpailurus_yaguarondi*	2	3	1	2	1	2	4	4	5	1	5	5	4	0	2	6	2
Felidae_*Leopardus_pardalis*	2	3	1	2	1	2	4	4	5	1	5	5	4	0	2	6	3
Felidae_*Leopardus_tigrinus*	2	4	1	2	1	3	4	4	4	1	5	5	4	0	2	6	2
Felidae_*Leopardus_wiedii*	2	3	1	2	1	2	4	4	5	1	5	5	4	0	2	6	2
Felidae_*Leptailurus_serval*	2	2	1	2	1	3	4	4	5	1	5	5	4	0	2	6	3
Felidae_*Lynx_canadensis*	2	2	1	2	1	2	4	4	5	1	5	5	4	0	2	6	3
Felidae_*Lynx_pardinus*	2	2	1	2	1	2	4	4	5	1	5	5	4	0	2	6	3
Felidae_*Lynx_rufus*	2	1	1	2	1	2	4	4	5	1	5	5	4	0	2	6	3
Felidae_*Lynx_lynx*	2	2	1	2	1	2	4	4	4	1	5	5	4	0	2	6	3
Felidae_*Neofelis_nebulosa*	2	2	1	2	1	2	4	4	5	1	5	5	4	0	2	6	3
Felidae_*Oncifelis_colocolo*	2	3	1	1	1	2	4	4	5	1	5	5	4	0	2	6	2
Felidae_*Oncifelis_geoffroyi*	2	3	1	2	1	2	4	4	5	1	5	5	4	0	2	6	2
Felidae_*Oncifelis_guigna*	2	2	1	2	1	3	4	4	4	1	5	5	4	0	2	6	2
Felidae_*Otocolobus_manul*	2	3	1	2	1	2	4	4	5	1	5	5	4	0	2	6	2
Felidae_*Panthera_leo*	2	3	1	2	1	2	4	4	5	1	5	5	4	0	2	6	6
Felidae_*Panthera_pardus*	2	2	1	2	1	2	4	4	5	1	5	5	4	0	2	6	5
Felidae_*Panthera_tigris*	2	3	1	2	1	2	4	4	5	1	5	5	4	0	2	6	6
Felidae_*Panthera_onca*	2	1	1	2	1	2	4	4	5	1	5	5	4	0	2	6	5
Felidae_*Pardofelis_marmorata*	2	3	1	2	1	2	4	4	5	1	5	5	4	0	2	6	2
Felidae_*Prionailurus_bengalensis*	2	3	1	2	1	3	4	4	5	1	5	5	4	0	2	6	2
Felidae_*Prionailurus_planiceps*	2	3	2	3	1	3	4	4	5	1	5	5	4	0	2	6	2
Felidae_*Prionailurus_rubiginosus*	2	2	1	2	1	2	4	4	5	1	5	5	4	0	2	6	1
Felidae_*Prionailurus_viverrinus*	2	3	1	2	1	2	4	4	5	1	5	5	4	0	2	6	3
Felidae_*Profelis_aurata*	2	2	1	2	1	3	4	4	5	1	5	5	4	0	2	6	3
Felidae_*Puma_concolor*	2	2	1	2	1	2	4	4	5	1	5	5	4	0	2	6	5

Felidae_*Uncia_uncia*	2	2	1	1	1	2	4	4	5	1	5	5	4	0	2	6	4
Herpestidae_*Atilax_paludinosus*	2	3	2	1	1	2	3	3	3	3	3	3	2	2	2	3	2
Herpestidae_*Bdeogale_crassicauda*	1	3	3	1	1	1	2	2	3	3	2	2	2	2	2	2	2
Herpestidae_*Bdeogale_jacksoni*	1	4	2	1	2	2	1	2	3	3	2	2	2	2	2	2	2
Herpestidae_*Bdeogale_nigripes*	1	4	2	1	1	2	1	2	2	4	1	2	2	2	2	2	2
Herpestidae_*Crossarchus_alexandri*	1	4	2	1	2	1	3	2	2	3	3	2	2	2	2	2	1
Herpestidae_*Crossarchus_obscurus*	1	4	2	1	1	1	3	3	3	3	2	3	2	2	2	2	1
Herpestidae_*Cynictis_penicillata*	1	4	2	1	1	2	3	3	2	2	3	3	3	2	2	3	1
Herpestidae_*Dologale_dybowskii*	1	3	2	1	1	2	3	3	2	3	4	2	2	2	2	2	1
Herpestidae_*Galerella_pulverulenta*	2	3	2	1	1	2	4	3	3	2	3	3	3	1	2	3	1
Herpestidae_*Galerella_sanguinea*	2	3	2	1	2	2	4	3	3	1	4	3	3	1	2	3	1
Herpestidae_*Helogale_hirtula*	1	4	2	1	1	1	3	3	3	3	3	2	3	2	2	2	1
Herpestidae_*Helogale_parvula*	1	5	2	1	1	1	2	3	2	3	3	2	3	2	2	2	1
Herpestidae_*Herpestes_ichneumon*	2	3	2	1	2	2	4	4	3	2	3	3	3	1	2	3	3
Herpestidae_*Ichneumia_albicauda*	1	4	3	1	2	2	3	3	3	3	2	2	2	2	2	2	2
Herpestidae_*Liberiictis_kuhni*	1	4	3	1	2	2	3	2	2	4	2	2	2	2	2	2	2
Herpestidae_*Mungos_gambianus*	1	3	2	1	1	1	2	2	2	4	2	3	3	2	2	2	1
Herpestidae_*Mungos_mungo*	1	4	2	1	1	1	2	2	2	3	2	2	3	2	2	2	2
Herpestidae_*Paracynictis_selousi*	2	3	3	1	2	2	3	2	3	2	2	3	3	2	2	2	2
Herpestidae_*Rhynchogale_melleri*	1	3	2	1	2	1	2	1	3	3	2	2	2	2	2	2	2
Herpestidae_*Suricata_suricata*	1	3	2	1	1	1	2	2	2	3	3	2	3	2	2	2	1
Herpestidae_*Urva_brachyurus*	2	4	2	1	1	2	4	3	3	2	3	3	3	1	2	3	2
Herpestidae_*Urva_edwardsii*	2	3	2	1	2	2	4	4	3	1	3	3	3	1	2	4	1
Herpestidae_*Urva_javanicus*	2	2	2	1	2	2	4	4	3	2	4	3	3	1	2	3	1
Herpestidae_*Urva_semitorquatus*	2	3	2	1	1	2	4	3	3	2	3	3	2	1	2	3	2

Herpestidae_*Urva_smithii*	2	2	2	1	1	2	4	4	3	2	3	3	3	1	2	3	2
Herpestidae_*Urva_urva*	2	3	2	1	2	2	4	3	3	2	3	3	3	1	2	3	2
Herpestidae_*Urva_vitticollis*	2	3	2	1	1	2	3	3	2	2	2	3	3	1	2	2	2
Hyaenidae_*Crocuta_crocuta*	2	3	3	1	1	2	4	4	5	1	5	5	4	0	2	6	5
Hyaenidae_*Hyaena_hyaena*	1	3	3	1	1	2	4	4	3	3	4	4	4	0	2	5	4
Hyaenidae_*Parahyaena_brunnea*	2	2	1	1	2	1	4	4	4	1	4	5	4	0	2	6	4
Hyaenidae_*Proteles_cristatus*	1	3	2	1	2	2	1	1	1	4	3	4	4	0	1	6	3
Mephitidae_*Conepatus_chinga*	1	3	1	1	1	1	3	4	2	3	2	3	1	1	2	2	1
Mephitidae_*Conepatus_humboldtii*	1	2	1	1	1	1	3	4	2	3	2	3	1	1	2	2	1
Mephitidae_*Conepatus_leuconotus*	2	3	1	1	1	1	3	4	2	3	1	3	1	1	2	2	2
Mephitidae_*Conepatus_mesoleucus*	2	2	1	1	1	1	3	4	2	3	1	3	1	1	2	2	2
Mephitidae_*Conepatus_semistriatus*	1	3	1	1	1	1	3	4	2	3	2	3	1	1	2	2	2
Mephitidae_*Mephitis_macroura*	2	3	1	2	1	1	3	4	3	3	2	3	2	1	2	2	1
Mephitidae_*Mephitis_mephitis*	1	2	2	1	1	1	3	4	3	3	2	3	1	1	2	3	2
Mephitidae_*Mydaus_javanensis*	1	5	2	2	2	2	1	5	3	4	1	3	1	1	2	2	2
Mephitidae_*Mydaus_marchei*	1	2	2	1	2	1	1	5	2	4	1	3	1	1	2	3	2
Mephitidae_*Spilogale_putorius*	1	3	2	2	1	1	3	4	3	3	3	3	1	1	2	3	1
Mephitidae_*Spilogale_pygmaea*	1	3	1	2	1	1	3	5	3	3	2	3	1	1	2	3	1
Mustelidae_*Amblonyx_cinereus*	2	1	2	1	1	1	2	4	3	3	2	3	2	1	2	3	2
Mustelidae_*Aonyx_capensis*	2	1	2	1	1	1	1	4	3	4	1	3	2	1	2	3	3
Mustelidae_*Aonyx_congicus*	2	1	2	1	1	1	2	3	3	4	1	3	2	1	2	3	4
Mustelidae_*Arctonyx_collaris*	1	4	2	2	2	2	2	1	3	3	1	4	1	1	2	3	2
Mustelidae_*Eira_barbara*	1	2	2	1	1	2	4	4	4	3	3	4	2	1	2	4	2
Mustelidae_*Enhydra_lutris*	2	1	1	1	1	1	2	1	3	4	1	3	2	1	1	1	4
Mustelidae_*Galictis_cuja*	2	2	1	1	1	1	3	4	3	3	2	3	2	1	2	3	2

Mustelidae_*Galictis_vittata*	1	2	2	1	1	1	3	4	4	2	3	4	2	1	2	4	2
Mustelidae_*Gulo_gulo*	2	2	3	1	1	1	4	4	4	1	3	5	2	1	2	4	3
Mustelidae_*Ictonyx_libyca*	2	2	2	2	1	2	4	4	3	2	2	3	2	1	2	3	1
Mustelidae_*Ictonyx_striatus*	2	3	2	2	1	2	4	4	3	2	3	3	2	1	2	4	1
Mustelidae_*Lontra_canadensis*	2	3	2	1	1	2	1	4	3	3	2	3	2	1	2	2	3
Mustelidae_*Lontra_felina*	2	1	2	1	1	1	1	4	3	3	2	3	2	1	2	3	4
Mustelidae_*Lontra_longicaudis*	2	1	2	1	1	1	1	4	3	3	2	3	2	1	2	3	2
Mustelidae_*Lontra_provocax*	2	1	2	1	1	1	1	4	3	3	1	3	2	1	2	6	3
Mustelidae_*Lutra_lutra*	2	1	2	1	1	2	3	4	3	3	2	3	2	1	2	3	3
Mustelidae_*Lutra_maculicollis*	2	1	2	1	1	1	3	4	3	3	2	3	2	1	2	3	2
Mustelidae_*Lutra_sumatrana*	2	2	2	2	1	2	3	4	3	3	3	3	2	1	2	3	2
Mustelidae_*Lutrogale_perspicillata*	2	2	2	1	1	2	1	4	3	4	2	3	2	1	2	3	3
Mustelidae_*Martes_americana*	2	2	3	2	1	2	4	4	3	2	3	4	2	1	2	3	1
Mustelidae_*Martes_flavigula*	2	3	2	2	1	2	4	4	3	2	3	3	2	1	2	4	2
Mustelidae_*Martes_foina*	2	2	2	2	1	2	4	4	4	1	3	4	2	1	2	4	1
Mustelidae_*Martes_martes*	2	3	2	2	1	2	4	4	3	2	3	4	2	1	2	3	1
Mustelidae_*Martes_melampus*	2	3	2	2	1	2	4	4	3	2	2	4	2	1	2	4	1
Mustelidae_*Martes_pennanti*	2	3	3	2	1	2	4	4	3	1	3	4	2	1	2	4	2
Mustelidae_*Martes_zibellina*	2	3	2	2	1	2	4	4	3	2	2	4	2	1	2	3	1
Mustelidae_*Meles_meles*	1	2	2	1	1	2	2	4	3	3	1	4	1	1	2	2	3
Mustelidae_*Mellivora_capensis*	2	2	2	1	1	1	4	4	4	1	3	5	2	1	2	5	3
Mustelidae_*Melogale_everetti*	1	3	2	1	1	2	3	4	3	2	2	3	2	1	2	3	2
Mustelidae_*Melogale_moschata*	1	3	3	1	2	2	3	4	3	2	3	3	2	1	2	3	1
Mustelidae_*Melogale_orientalis*	2	4	2	1	1	2	1	4	3	2	2	3	2	1	2	3	1
Mustelidae_*Melogale_personata*	1	3	2	1	1	2	3	4	3	3	2	3	2	1	2	3	2

Mustelidae_*Mustela_africana*	2	2	2	2	1	2	4	4	3	1	2	5	2	1	2	4	1
Mustelidae_*Mustela_altaica*	2	2	2	3	2	2	4	4	3	1	3	5	2	1	2	4	1
Mustelidae_*Mustela_erminea*	2	2	2	2	1	2	4	4	3	1	2	5	2	1	2	4	1
Mustelidae_*Mustela_eversmannii*	2	3	2	2	1	2	4	4	4	1	3	5	2	1	2	4	1
Mustelidae_*Mustela_felipei*	2	1	2	2	1	2	4	4	3	1	3	5	2	1	2	4	1
Mustelidae_*Mustela_frenata*	2	2	2	2	1	2	4	4	3	2	2	5	2	1	2	4	1
Mustelidae_*Mustela_kathiah*	2	3	2	3	2	2	4	4	3	2	2	5	2	1	2	4	1
Mustelidae_*Mustela_lutreola*	2	2	2	2	2	2	4	4	3	1	2	5	2	1	2	4	1
Mustelidae_*Mustela_lutreolina*	2	2	2	3	2	2	4	4	4	1	3	5	2	1	2	4	1
Mustelidae_*Mustela_nigripes*	2	2	2	1	2	1	4	4	4	1	3	5	2	1	2	4	1
Mustelidae_*Mustela_nivalis*	2	2	2	2	2	2	4	4	3	1	3	5	2	1	2	4	1
Mustelidae_*Mustela_nudipes*	2	2	2	2	1	3	4	4	3	2	3	5	2	1	2	4	1
Mustelidae_*Mustela_putorius*	2	3	2	2	1	2	4	4	3	2	2	5	2	1	2	4	1
Mustelidae_*Mustela_sibirica*	2	4	2	2	1	2	4	4	4	1	3	5	2	1	2	4	1
Mustelidae_*Mustela_vison*	2	2	2	2	1	2	4	4	3	3	2	5	2	1	2	4	1
Mustelidae_*Poecilogale_albinucha*	2	2	1	2	1	2	4	4	3	1	3	5	2	1	2	5	1
Mustelidae_*Pteronura_brasiliensis*	2	2	2	1	1	2	1	4	3	3	2	3	2	1	2	3	4
Mustelidae_*Taxidea_taxus*	2	2	2	1	1	2	1	3	3	3	3	4	1	1	2	3	3
Mustelidae_*Vormela_peregusna*	2	2	1	2	1	2	3	4	3	2	3	3	2	1	2	4	1
Nandiniidae_*Nandinia_binotata*	2	4	3	1	2	1	4	4	4	1	3	3	3	1	2	4	2
Prionodontidae_*Prionodon_linsang*	2	4	3	3	2	3	4	4	4	1	4	4	3	1	2	4	1
Prionodontidae_*Prionodon_pardicolor*	2	3	3	3	2	3	4	4	4	1	4	4	3	1	2	5	1
Procyonidae_*Bassaricyon_alleni*	1	1	3	1	2	1	2	1	2	3	1	2	1	2	1	1	1
Procyonidae_*Bassaricyon_gabbi*	1	2	3	1	2	1	2	1	2	4	1	2	1	2	1	1	1
Procyonidae_*Bassaricyon_pauli*	1	2	3	1	2	1	2	1	2	4	1	2	1	2	1	1	1

Procyonidae_*Bassariscus_astutus*	2	3	3	2	2	2	3	3	3	1	3	3	2	2	2	3	1
Procyonidae_*Bassariscus_sumichrasti*	2	2	3	1	2	1	3	3	3	3	3	3	2	2	2	2	1
Procyonidae_*Nasua_narica*	1	5	3	1	2	1	2	1	2	4	2	3	1	2	2	1	2
Procyonidae_*Nasua_nasua*	1	4	3	1	2	1	2	1	2	3	1	2	1	2	2	1	2
Procyonidae_*Nasuella_olivacea*	1	5	3	2	2	2	2	1	2	3	1	3	1	2	2	1	2
Procyonidae_*Potos_flavus*	1	3	2	1	1	1	1	1	1	4	2	1	1	2	2	1	2
Procyonidae_*Procyon_cancrivorus*	1	3	3	1	1	1	1	1	3	3	1	3	1	2	1	1	2
Procyonidae_*Procyon_gloveralleni*	1	3	3	1	2	1	1	1	3	3	1	3	1	2	2	1	2
Procyonidae_*Procyon_insularis*	1	2	3	1	1	1	1	1	3	3	1	3	1	2	2	1	2
Procyonidae_*Procyon_lotor*	1	3	3	1	1	1	1	1	3	3	1	3	1	2	2	1	2
Procyonidae_*Procyon_maynardi*	1	3	3	1	2	1	1	1	3	3	1	3	1	2	2	1	2
Procyonidae_*Procyon_pygmaeus*	1	2	3	1	2	1	1	1	3	3	1	3	1	2	2	1	2
Ursidae_*Ailuropoda_melanoleuca*	2	3	2	2	1	2	1	1	3	4	1	3	1	2	2	1	6
Ursidae_*Helarctos_malayanus*	1	3	3	1	2	2	2	4	3	4	1	4	1	2	2	2	5
Ursidae_*Melursus_ursinus*	1	3	0	1	2	2	2	4	3	4	1	4	1	2	2	2	5
Ursidae_*Tremarctos_ornatus*	1	4	1	1	1	1	2	4	3	4	1	4	1	2	2	2	6
Ursidae_*Ursus_americanus*	1	3	0	1	1	2	2	2	3	4	2	3	1	2	2	1	5
Ursidae_*Ursus_arctos*	1	2	0	1	1	1	3	2	2	4	2	2	1	2	2	1	6
Ursidae_*Ursus_thibetanus*	1	3	0	1	1	1	3	2	3	4	1	4	1	2	2	2	5
Ursidae_*Ursus_maritimus*	2	2	0	1	1	1	3	2	3	4	1	4	1	2	2	2	6
Viverridae_*Arctictis_binturong*	1	4	3	1	2	1	2	1	1	3	2	3	1	1	1	4	3
Viverridae_*Arctogalidia_trivirgata*	2	4	3	1	2	1	2	1	3	4	1	3	1	2	2	3	2
Viverridae_*Civettictis_civetta*	1	2	3	1	2	2	3	3	3	2	2	3	2	2	1	2	3
Viverridae_*Cynogale_bennettii*	2	4	3	3	2	3	2	2	2	4	1	3	1	2	2	2	2
Viverridae_*Diplogale_hosei*	2	4	3	2	2	3	3	3	2	3	2	3	3	2	2	2	2

Viverridae_*Genetta_abyssinica*	1	4	3	2	2	3	4	3	3	1	3	3	3	1	2	3	2
Viverridae_*Genetta_angolensis*	2	2	2	2	2	3	4	4	3	2	3	3	3	1	2	3	2
Viverridae_*Genetta_genetta*	2	2	3	2	2	3	4	3	3	3	3	3	3	2	2	3	2
Viverridae_*Genetta_maculata*	2	2	2	2	2	3	4	3	3	2	3	3	3	1	2	3	1
Viverridae_*Genetta_servalina*	2	3	2	2	2	3	4	3	3	2	3	3	3	1	2	3	1
Viverridae_*Genetta_thierryi*	2	2	3	1	2	2	4	4	3	2	3	3	3	1	2	3	1
Viverridae_*Genetta_tigrina*	2	2	2	2	2	3	4	3	3	2	4	3	3	1	2	4	2
Viverridae_*Genetta_victoriae*	1	3	2	2	2	2	4	3	3	2	3	3	3	1	2	3	2
Viverridae_*Hemigalus_derbyanus*	1	4	3	1	2	3	3	2	2	3	1	2	1	2	2	2	2
Viverridae_*Paguma_larvata*	1	4	2	1	2	1	3	3	3	3	1	3	2	1	1	3	2
Viverridae_*Paradoxurus_hermaphroditus*	1	3	3	1	2	1	3	3	3	3	2	3	2	1	1	1	2
Viverridae_*Paradoxurus_jerdoni*	1	3	3	1	2	1	3	3	2	3	1	3	2	1	1	1	2
Viverridae_*Paradoxurus_zeylonensis*	1	3	3	1	2	1	3	3	3	3	1	3	2	1	1	1	2
Viverridae_*Poiana_richardsonii*	2	3	2	2	2	3	4	4	3	2	4	4	3	1	2	4	1
Viverridae_*Viverra_megaspila*	1	3	3	2	2	3	4	3	3	2	3	3	2	2	2	3	3
Viverridae_*Viverra_tangalunga*	1	3	3	2	2	2	4	4	2	1	3	3	3	2	2	2	2
Viverridae_*Viverra_zibetha*	1	4	3	2	2	2	4	3	3	2	3	3	2	2	2	3	3
Viverridae_*Viverricula_indica*	1	2	3	2	2	2	4	3	3	2	3	3	2	2	2	3	2

[1]Coded based on *H. inexpecta* and *H. parvula* (see table S4).

Table S8. Allocations of South American Land Mammal Ages (SALMAs) and temporally distinct faunas to 2-million-year bins. Bin number corresponds to numbering system used by Wesley-Hunt [52] for North American carnivoramorphans. Names in brackets are faunas that have been suggested to represent new SALMAs but have not yet been formally named. Bins not represented by a SALMA are indicated by a hyphen. Absolute ages of SALMAs are primarily based on Flynn and Swisher [62], as modified by Gelfo *et al.* [63],[64],López [65],Ré *et al.* [66],Dunn *et al.* [67]. The Santa Rosa Fauna [68,69] is considered to be Tinguiririan in age in the absence of independent age constraints. The Friasian SALMA sensu stricto was excluded because it includes no unique taxa relative to flanking intervals (Santacrucian and Colloncuran), and its temporal relationships are unclear [70,71]. OTU occurrences by interval are listed in table S1. Abbreviations: Ma, megannum; OTU, operational taxonomic unit.

**Bin Number**	**Bin age (Ma)**	**SALMA or informal unit**	**Source(s) for OTU occurrence data**
1	2-0	-	-
2	4-2	Chapadmalalan[1]	[1]
3	6-4	Montehermosan[2]	[1]
4	8-6	Huayquerian	[1]
5	10-8	Chasicoan	[1]
6	12-10	Mayoan	(none)
7	14-12	Laventan	[1,15,40]
8	16-14	Colloncuran	[1,31,37]

9	18-16	Santacrucian	[1]
10	20-18	-	-
11	22-20	Colhuehuapian	[1,38]
12	24-22	-	-
13	26-24	Deseadan	[1,8]
14	28-26	Deseadan	(see above)
15	30-28	Deseadan	(see above)
16	32-30	[La Cantera]	[41]
17	34-32	Tinguiririican	[39]
18	36-34	-	-
19	38-36	Mustersan	[1]
20	40-38	-	-
21	42-40	Barrancan	[1,36]
22	44-42	Vacan	[64]
23	46-44	Riochican	[64]
24	48-46	-	-
25	50-48	[Laguna Fría]	[27,64]
26	52-50	-	-
27	54-52	-	-
28	56-54	Itaboraian	[64]
29	58-56	-	-

30	60-58	-	-

[1] Primarily spans early (early Pliocene) portion of interval

[2] Only spans latter (early Pliocene) portion of interval

Table S9. Species lists and diet categorizations for three modern carnivore guilds (Malaysia, the Serengeti, and Yellowstone National Park), the early Miocene of Santa Cruz (Argentina), and three biochrons of the White River Group (late Eocene to Oligocene, Central and Nothern Great Plains,United States). Species are listed alphabetically by guild. Abbreviations: Car, carnivorous; Ch3, Chadronian 3 of White River Group; Hypo, hypocarnivore; Hyper, hypercarnivore; Ins, insectivorous; M, Malaysia; MB, meat/bone; Meso, mesocarnivore; MNv, meat/nonvertebrate; NvM, nonvertebrate/meat; OHo, omnivorous/hard object; Or2, Orellan 2 of White River Group; PBDB, data from Paleobiology Database (https://paleobiodb.org; accessed Nov. 6, 2017); SC, Santa Cruz; Sg, Serengeti; Wh2, Whitneyan 2 of White River Group; Y, Yellowstone.

**Taxon**	**Family**	**Guild**	**Guild Source**	**Diet Source**	**Diet Original**	**Diet This Study**	**Notes**
Brachyrhynchocyon dodgei	Amphicyonidae	Ch3	[72,73], PBDB	[52,74]	-	Meso	
Daphoenictis tedfordi	Amphicyonidae	Ch3	[73,75]	[52,74]	-	Hyper	
Daphoenus hartshornianus	Amphicyonidae	Ch3	[72], PBDB	[52,74]	-	Hypo	
Daphoenus vetus	Amphicyonidae	Ch3	[72], PBDB	[52,74]	-	Hypo	
Dinictis felina	Nimravidae	Ch3	[76,77]	[52,74]	-	Hyper	

Hesperocyon gregarius	Canidae	Ch3	[78]	[74]	-	Meso	
Hoplophoneus cerebralis	Nimravidae	Ch3	[76,77]	[52,74]	-	Hyper	
Hoplophoneus oharrai	Nimravidae	Ch3	[76,77]	-	-	(Hyper)	Diet based on other *Hoplophoneus* spp.
Hoplophoneus primaevus	Nimravidae	Ch3	[76,77]	[52,74]	-	Hyper	
Hyaenodon crucians	Hyaenodontidae	Ch3	[73,78], PBDB	[74]	-	Hyper	
Hyaenodon horridus	Hyaenodontidae	Ch3	[73,78], PBDB	[74]	-	Hyper	
Hyaenodon megaloides	Hyaenodontidae	Ch3	[73]	[74]	-	Hyper	
Hyaenodon microdon	Hyaenodontidae	Ch3	[73]	[74]	-	Hyper	
Hyaenodon montanus	Hyaenodontidae	Ch3	[73], PBDB	[74]	-	Hyper	
Mustelavus priscus	Mustelidae	Ch3	PBDB		-	Meso	
Parictis dakotensis	Ursidae	Ch3	PBDB	[74]	-	Meso	
Parictis gilpini	Ursidae	Ch3	PBDB	[74]	-	Meso	
Arctictis binturong	Viverridae	M	[54]	[54]	NvM	Hypo	

Arctogalidia trivirgata	Viverridae	M	[79] (Krau)	[80]	-	Hypo	
Arctonyx collaris	Mustelidae	M	[79] (Cat Tien)	[81]	-	Hypo	
Catopuma temminickii	Felidae	M	[54]	[54]	Meat	Hyper	
Cuon alpinus	Canidae	M	[54]	[54]	Meat	Hyper	
Cynogale bennettii	Viverridae	M	[79] (Krau)	[82]	OHo	Hypo	
Hemigalus derbyanus	Viverridae	M	[79] (Krau)	[82]	OHo	Hypo	
Martes flavigula	Mustelidae	M	[79] (Krau)	([82])	(Car)	Hyper	Diet based on other *Martes* spp.
Melogale personata	Mustelidae	M	[79] (Cat Tien)	([82])	(OHo)	Hypo	Diet based on *M. moschata*
Mustela nudipes	Mustelidae	M	[79] (Krau)	([82])	(Car)	Hyper	Diet based on other *Mustela* spp.
Neofelis nebulosa	Felidae	M	[54]	[54]	Meat	Hyper	
Paguma larvata	Viverridae	M	[79] (Krau)	[82]	OHo	Hypo	
Panthera pardus	Felidae	M	[54]	[54]	Meat	Hyper	
Panthera tigris	Felidae	M	[54]	[54]	Meat	Hyper	
Paradoxurus hermaphroditus	Viverridae	M	[79] (Krau)	[82]	OHo	Hypo	

Prionailurus bengalensis	Felidae	M	[79] (Krau)	([54])	(Meat)	Hyper	Diet based on other Felidae
Prionailurus planiceps	Felidae	M	[79] (Krau)	([54])	(Meat)	Hyper	Diet based on other Felidae
Prionailurus viverrinus	Felidae	M	[54]	[54]	Meat	Hyper	
Prionodon linsang	Prionodontidae	M	[79] (Krau)	[82]	Car	Hyper	Diet based on *P. pardicolor*
Prionodon pardicolor	Prionodontidae	M	[79] (Cat Tien)	[82]	Car	Hyper	
Ursus thibetanus	Ursidae	M	[54]	[54]	NvM	Hypo	
Urva brachyurus	Herpestidae	M	[79] (Krau)	([82])	(Car)	Hyper	Diet based on *U. javanicus*
Urva javanicus	Herpestidae	M	[79] (Cat Tien)	[82]	Car	Hyper	
Urva urva	Herpestidae	M	[79] (Cat Tien)	([82])	(Car)	Hyper	Diet based on *U. javanicus*
Viverra megaspila	Viverridae	M	[54]	[54]	MNv	Meso	
Viverra zibetha	Viverridae	M	[79] (Cat Tien)	[82]	Car	Hyper	
Viverricula indica	Viverridae	M	[79] (Krau)	[82]	Car	Hyper	
Daphoenus hartshornianus	Amphicyonidae	Or2	[72], PBDB	[52,74]	-	Hypo	
Daphoenus vetus	Amphicyonidae	Or2	[72], PBDB	[52,74]	-	Hypo	

Dinictis felina	Nimravidae	Or2	[76,77]	[52,74]	-	Hyper	
?Drassonax sp.	Ursidae	Or2	[72,73, 83]	-	-	Meso	Based on *Parictis*
"Hesperocyon" coloradensis	Canidae	Or2	[73,84]	[74]	-	Meso	*H. lippincottianus* in Van Valkenburgh [74]
Hesperocyon gregarius	Canidae	Or2	[78]	[74]	-	Meso	
Hoplophoneus occidentalis	Nimravidae	Or2	[76,77]	[52,74]	-	Hyper	
Hoplophoneus primaevus	Nimravidae	Or2	[76,77]	[52,74]	-	Hyper	
Hoplophoneus sicarius	Nimravidae	Or2	[76,77]	[52,74]	-	Hyper	
Hyaenodon crucians	Hyaenodontidae	Or2	[73,78], PBDB	[74]	-	Hyper	
Hyaenodon horridus	Hyaenodontidae	Or2	[73,78], PBDB	[74]	-	Hyper	
Leptocyon sp. A	Canidae	Or2	[85]	[52]	-	Hypo	
"Mesocyon" temnodon	Canidae	Or2	[84], PBDB	[52,74]	-	Meso	*"Mesocyon"* sp. in Van Valkenburgh [74]

Mustelavus priscus	Mustelidae	Or2	PBDB		-	Meso	
Nanosmilus kurteni	Nimravidae	Or2	[76,77]	-	-	Hyper	Diet based on *Hoplophoneus*
Otarocyon cooki	Canidae	Or2	[86]	[74]	-	Meso	
Palaeogale sectoria	Carnivora incertae sedis	Or2	PBDB	[74]	-	Hyper	
Paradaphoenus minimus	Amphicyonidae	Or2	[87]	[74]	-	Hypo	
Parictis dakotensis	Ursidae	Or2	PBDB	[74]	-	Meso	
Acrocyon sectorius	Borhyaenidae	SC	[88]	[3]	Hyper	Hyper	Hyper sensu Marshall [89]
Acyon tricuspidatus	Hathliacynidae	SC	[88]	[3]	Hyper	Hyper	Meso sensu Marshall [89]
Arctodictis munizi	Borhyaenidae	SC	[88]	[3]	Hyper	Hyper	Hyper sensu Marshall [89]
Borhyaena tuberata	Borhyaenidae	SC	[88]	[3]	Hyper	Hyper	Hyper sensu Marshall [89]
Cladosictis patagonica	Hathliacynidae	SC	[88]	[3]	Hyper	Hyper	Meso sensu Marshall [89]
Lycopsis torresi	Borhyaenoidea	SC	[88]	[3]	Hyper	Hyper	Meso sensu Marshall [89]

Patagornis marshi	Phorusrhacidae	SC	[90]	[90]	Hyper	Hyper	
Perathereutes pungens	Hathliacynidae	SC	[88]	[3]	Hyper	Hyper	Hypo sensu Marshall [89]
Phorusrhacos longissimus	Phorusrhacidae	SC	[90]	[90]	Hyper	Hyper	
Prothylacynus patagonicus	Borhyaenoidea	SC	[88]	[3]	Hyper	Hyper	Meso sensu Marshall [89]
Pseudonotictis pusillus	Hathliacynidae	SC	[88]	[3]	Hyper	Hyper	Hypo sensu Marshall [89]
Psilopterus bachmanni	Phorusrhacidae	SC	[90]	[90]	Hyper	Hyper	
Psilopterus lemoinei	Phorusrhacidae	SC	[90]	[90]	Hyper	Hyper	
Sipalocyon gracilis	Hathliacynidae	SC	[88]	[3]	Hyper	Hyper	Hypo sensu Marshall [89]
Sipalocyon obusta	Hathliacynidae	SC	[88]	[3]	Hyper	Hyper	Hypo sensu Marshall [89]
Acinonyx jubatus	Felidae	Sg	[54]	[54]	Meat	Hyper	
Atilax paludinosus	Herpestidae	Sg	[91]	[82]	OHo	Hypo	
Canis adustus	Canidae	Sg	[54]	[54]	MNv	Meso	
Canis aureus	Canidae	Sg	[54]	[54]	MNv	Meso	
Canis mesomelas	Canidae	Sg	[54]	[54]	MNv	Meso	
Caracal caracal	Felidae	Sg	[54]	[54]	Meat	Hyper	

Civetticus civetta	Viverridae	Sg	[54]	[54]	MNv	Meso	
Crocuta crocuta	Hyaenidae	Sg	[54]	[54]	MB	Hyper	
Felis silvestris lybica	Felidae	Sg	[91]	[54]	(Meat)	Hyper	Diet based on other Felidae
Galerella sanguinea	Herpestidae	Sg	[91]	[82]	Car	Hyper	
Genetta genetta	Viverridae	Sg	[91]	[82]	Car	Hyper	
Genetta tigrina	Viverridae	Sg	[91]	[82]	Car	Hyper	
Helogale parvula	Herpestidae	Sg	[91]	[82]	Ins	Hypo	
Herpestes ichneumon	Herpestidae	Sg	[91]	[82]	Car	Meso	
Hyaena hyaena	Hyaenidae	Sg	[54]	[54]	MB	Hyper	
Ichneumia albicauda	Herpestidae	Sg	[91]	[82]	Ins	Hypo	
Ictonyx striatus	Mustelidae	Sg	[91]	[82]	Car	Hyper	
Leptailurus serval	Felidae	Sg	[54]	[54]	Meat	Hyper	
Lycaon pictus	Canidae	Sg	[54]	[54]	Meat	Hyper	
Mellivora capensis	Mustelidae	Sg	[54]	[54]	MNv	Meso	
Mungos mungo	Herpestidae	Sg	[91]	[82]	Ins	Hypo	
Otocyon megalotis	Canidae	Sg	[91]	[80]	-	Hypo	
Panthera leo	Felidae	Sg	[54]	[54]	Meat	Hyper	
Panthera pardus	Felidae	Sg	[54]	[54]	Meat	Hyper	
Archaeocyon leptodus	Canidae	Wh2	[86]	[74]	-	Meso	

Archaeocyon pavidus	Canidae	Wh2	[86]	[74]	-	Meso	*"Nothocyon"* sp. 1 in Van Valkenburgh [74]
Cynodesmus thooides	Canidae	Wh2	[78,84, 92]	[74]	-	Meso	*Mesocyon sheffleri* in Van Valkenburgh [74]
Daphoenus hartshornianus	Amphicyonidae	Wh2	[72], PBDB	[52,74]	-	Hypo	
Daphoenus vetus	Amphicyonidae	Wh2	[72], PBDB	[52,74]	-	Hypo	
Dinictis felina	Nimravidae	Wh2	[76,77]	[52,74]	-	Hyper	
Ectopocynus antiquus	Canidae	Wh2	[78,92]	[52,93]	-	Hyper	
Hesperocyon gregarius	Canidae	Wh2	[78]	[74]	-	Meso	
Hoplophoneus cerebralis	Nimravidae	Wh2	[76,77]	[52,74]	-	Hyper	
Hoplophoneus dakotensis	Nimravidae	Wh2	[76,77]	[52,74]	-	Hyper	
Hoplophoneus occidentalis	Nimravidae	Wh2	[76,77]	[52,74]	-	Hyper	
Hoplophoneus primaevus	Nimravidae	Wh2	[76,77]	[52,74]	-	Hyper	

Hyaenodon brevirostris	Hyaenodontidae	Wh2	[73]	[74]	-	Hyper	
"Mesocyon" temnodon	Canidae	Wh2	[84]	[52,74]	-	Meso	*“Mesocyon”* sp. in Van Valkenburgh [74]
Nimravus brachyops	Nimravidae	Wh2	[76,77]	[74]			
Osbornodon sesnoni	Canidae	Wh2	[84], PBDB	[52,74]	-	Meso	*Brachyrhynchocyon sesnoni* in Van Valkenburgh [74]
Oxetocyon cuspidatus	Canidae	Wh2	[78,86, 92]	[74]	-	Hypo	
Paradaphoenus tooheyi	Amphicyonidae	Wh2	[87]	[74]	-	Hypo	
Pogonodon davisi	Nimravidae	Wh2	[76,77]	[52,74]	-	Hyper	
Pogonodon platycopsis	Nimravidae	Wh2	[76,77]	[52,74]	-	Hyper	
Canis latrans	Canidae	Y	[54]	[54]	MNv	Meso	
Canis lupus	Canidae	Y	[54]	[54]	Meat	Hyper	
Gulo gulo	Mustelidae	Y	[54]	[54]	MNv	Meso	
Lynx canadensis	Felidae	Y	[54]	[54]	Meat	Hyper	
Lynx rufus	Felidae	Y	[54]	[54]	Meat	Hyper	
Martes americana	Mustelidae	Y	[79]	[82]	Car	Hyper	

Martes pennanti	Mustelidae	Y	[79]	[82]	Car	Hyper	
Mephitis mephitis	Mephitidae	Y	[79]	[82]	(OHo)	Hypo	Diet based on other Mephitidae
Mustela erminea	Mustelidae	Y	[79]	[82]	Car	Hyper	
Mustela frenata	Mustelidae	Y	[79]	[82]	Car	Hyper	
Mustela vison	Mustelidae	Y	[79]	[82]	Car	Hyper	
Procyon lotor	Procyonidae	Y	[79]	[82]	OHo	Hypo	
Puma concolor	Felidae	Y	[54]	[54]	Meat	Hyper	
Taxidea taxus	Mustelidae	Y	[54]	[54]	MNv	Meso	
Ursus americanus	Ursidae	Y	[54]	[54]	NvM	Hypo	
Ursus arctos	Ursidae	Y	[54]	[54]	NvM	Hypo	
Vulpes vulpes	Canidae	Y	[54]	[54]	MNv	Meso	

Table S10. Character loadings, eigenvalues, and % of variation (Vx) for first two axes of canonical analysis of all taxa (N = 361). Abbreviations for characters plotted in figure 2 are noted in parentheses, with corresponding values italicized.

**Character**	**Axis 1**	**Axis 2**
1	-0.184128	-0.496979
2	0.795109	0.67651
3 (PNo)	*1.15203*	*1.23184*
4 (PSh)	*-0.0121179*	*1.21439*
5 (PSp)	*1.0659*	*1.04174*
6	-0.00494292	0.934078
7	-0.38152	0.858246
8	-0.295147	0.212226
9	-0.425256	-0.427926
10 (CarOA)	*2.59578*	*-2.0038*
11 (CarBA)	*-1.06742*	*0.132848*
12	-0.442421	-0.299921
13	-0.955216	0.0236275
14 (MNo)	*2.58537*	*0.954987*
15	0.383284	0.145892
16 (LGA)	*-1.35588*	*-0.474978*
17 (BM)	*0.179953*	*-2.64991*
Eigenvalue	0.042771	0.0195834
% of Vx	37.949	17.376

Table S11. Taxon canonical analysis scores. Abbreviations: CA, canonical axis; Did, Didelphimorphia; Dsy, Dasyuromorphia; MC, modern Carnivora; NAC, North American Cenozoic carnivoramorphans; Sp, Sparassodonta.

**Taxon**	**Data Set**	**Family**	**CA 1**	**CA 2**
Borhyaenidae_*Acrocyon*	Sp	Borhyaenidae	-1.02675	-0.327934
Borhyaenidae_*Arctodictis*	Sp	Borhyaenidae	-1.02692	-1.02466
Borhyaenidae_*Australohyaena*	Sp	Borhyaenidae	-0.943705	-1.17746
Borhyaenidae_*Borhyaena*	Sp	Borhyaenidae	-0.609294	-0.822058
Borhyaenidae_indet	Sp	Borhyaenidae	-0.20253	-0.685362
Borhyaenoidea_*Angelocabrerus*	Sp	Borhyaenoidea	-0.654988	-0.972573
Borhyaenoidea_*Dukecynus*	Sp	Borhyaenoidea	-0.0616338	-1.25567
Borhyaenoidea_*Fredszalaya*	Sp	Borhyaenoidea	-0.342568	-0.041165
Borhyaenoidea_*Lycopsis*	Sp	Borhyaenoidea	-0.621052	-0.0257751
Borhyaenoidea_IGM251108	Sp	Borhyaenoidea	0.263028	0.342658
Borhyaenoidea_*Pharsophorus*	Sp	Borhyaenoidea	-0.786874	-0.654848
Borhyaenoidea_*Plesiofelis*	Sp	Borhyaenoidea	-0.457216	-0.761387
Borhyaenoidea_*Prothylacynus*	Sp	Borhyaenoidea	-0.717203	-0.354596
Borhyaenoidea_*Pseudolycopsis*	Sp	Borhyaenoidea	0.19766	-0.149879
Borhyaenoidea_*Pseudothylacynus*	Sp	Borhyaenoidea	-0.651159	-0.582692
Hathliacynidae_*Acyon*	Sp	Hathliacynidae	-0.604107	0.180988
Hathliacynidae_*Borhyaenidium*	Sp	Hathliacynidae	-0.508818	0.600555
Hathliacynidae_*Chasicostylus*	Sp	Hathliacynidae	-0.612904	0.11429
Hathliacynidae_*Cladosictis*	Sp	Hathliacynidae	-0.685775	0.539138

Hathliacynidae_MPEFPV4770	Sp	Hathliacynidae	-0.356327	0.32894
Hathliacynidae_*Notictis*	Sp	Hathliacynidae	-0.0337017	0.733421
Hathliacynidae_*Notocynus*	Sp	Hathliacynidae	-0.0982222	0.171314
Hathliacynidae_*Notogale*	Sp	Hathliacynidae	0.00358372	0.545667
Hathliacynidae_*Perathereutes*	Sp	Hathliacynidae	-0.311118	0.578935
Hathliacynidae_*Pseudonotictis*	Sp	Hathliacynidae	-0.582859	0.890295
Hathliacynidae_*Sallacyon*	Sp	Hathliacynidae	-0.491244	0.387504
Hathliacynidae_*Sipalocyon*	Sp	Hathliacynidae	-0.477939	0.732711
Hondadelphidae_Hondadelphys	Sp	Hondadelphidae	0.0547405	0.601699
Proborhyaenidae_*Arminiheringia*	Sp	Proborhyaenidae	-0.867815	-0.48925
Proborhyaenidae_*Callistoe*	Sp	Proborhyaenidae	-0.866888	-0.497535
Proborhyaenidae_*Paraborhyaena*	Sp	Proborhyaenidae	-0.87684	-1.19624
Proborhyaenidae_*Proborhyaena*	Sp	Proborhyaenidae	-0.621456	-1.09278
Sparassodonta_Genetspnov6	Sp	Sparassodonta	-0.0151785	0.689175
Sparassodonta_*Nemolestes*	Sp	Sparassodonta	7.29E-05	0.289159
Sparassodonta_*Patene*	Sp	Sparassodonta	-0.362603	0.771411
Sparassodonta_*Procladosictis*	Sp	Sparassodonta	-0.26939	0.156809
Sparassodonta_*Stylocynus*	Sp	Sparassodonta	0.136828	-0.409415
Sparassodonta_UF27881	Sp	Sparassodonta	-0.156118	0.676199
Thylacosmilidae_*Anachlysictis*	Sp	Thylacosmilidae	-0.222509	-1.12885
Thylacosmilidae_*Patagosmilus*	Sp	Thylacosmilidae	-0.135196	-0.441012
Thylacosmilidae_*Thylacosmilus*	Sp	Thylacosmilidae	-0.867083	-0.677245
Dasyuridae_*Dasyurus*	Dsy	Dasyuridae	-0.627887	0.395611
Dasyuridae_*Sarcophilus*	Dsy	Dasyuridae	-1.13384	-0.59417
Thylacinidae_*Thylacinus*	Dsy	Thylacinidae	-0.42146	-0.139532

Didelphidae_*Didelphis*	Did	Didelphidae	-0.0259483	1.36164
Didelphidae_*Hyperdidelphys*	Did	Didelphidae	-0.441748	0.992742
Didelphidae_*Lutreolina*	Did	Didelphidae	-0.0741913	1.50942
Didelphidae_*Thylatheridium*	Did	Didelphidae	-0.563156	0.777726
Didelphidae_*Thylophorops*	Did	Didelphidae	-0.325264	0.932658
Sparassocynidae_*Sparassocynus*	Did	Sparassocynidae	-0.0529048	0.899639
Amphicyonidae_*Amphicyon*	NAC	Amphicyonidae	0.423127	-0.429523
Amphicyonidae_*Brachyrhynchocyon*	NAC	Amphicyonidae	0.0351408	0.56537
Amphicyonidae_*Daphoenodon*	NAC	Amphicyonidae	0.552614	-0.483168
Amphicyonidae_*Daphoenus*	NAC	Amphicyonidae	1.00833	0.351634
Amphicyonidae_*Ischyrocyon*	NAC	Amphicyonidae	0.455766	-1.15565
Amphicyonidae_*Paradaphoenus*	NAC	Amphicyonidae	0.436559	1.4133
Amphicyonidae_*Pliocyon*	NAC	Amphicyonidae	0.659934	-0.994422
Amphicyonidae_*Temnocyon*	NAC	Amphicyonidae	0.850075	-0.18299
Barbourofelidae_*Barbourofelis*	NAC	Barbourofelidae	-1.27902	-0.821101
Canidae_*Aelurodon*	NAC	Canidae	-0.376681	0.154794
Canidae_*Borophagus*	NAC	Canidae	-0.249649	0.153196
Canidae_*Caedocyon*	NAC	Canidae	0.23335	-0.0760845
Canidae_*Canis_2*	NAC	Canidae	0.289105	0.849963
Canidae_*Carpocyon*	NAC	Canidae	0.149695	0.332139
Canidae_*Cerdocyon*	NAC	Canidae	0.31843	1.10113
Canidae_*Chrysocyon*	NAC	Canidae	0.444678	0.946419
Canidae_*Cormocyon*	NAC	Canidae	0.320597	1.2096
Canidae_*Cynarctoides*	NAC	Canidae	0.351019	1.11857
Canidae_*Cynarctus*	NAC	Canidae	0.739241	0.949702

Canidae_*Cynodesmus*	NAC	Canidae	0.00396496	0.50323
Canidae_*Ectopocynus*	NAC	Canidae	-0.468318	0.0233805
Canidae_*Enhydrocyon*	NAC	Canidae	-0.701458	-0.347549
Canidae_*Epicyon*	NAC	Canidae	-0.197536	-0.12525
Canidae_*Euoplocyon*	NAC	Canidae	-0.434159	0.249719
Canidae_*Hesperocyon*	NAC	Canidae	0.323976	1.50005
Canidae_*Leptocyon*	NAC	Canidae	0.344818	1.4516
Canidae_*Mesocyon*	NAC	Canidae	0.203138	0.890864
Canidae_*Osbornodon*_1	NAC	Canidae	0.0747857	1.06919
Canidae_*Osbornodon*_2	NAC	Canidae	0.0471689	0.184219
Canidae_*Otarocyon*	NAC	Canidae	0.0703205	1.06473
Canidae_*Paracynarctus*	NAC	Canidae	0.707529	0.320378
Canidae_*Paraenhydrocyon*	NAC	Canidae	0.208298	0.75941
Canidae_*Paratomarctus*	NAC	Canidae	0.125082	0.577187
Canidae_*Philotrox*	NAC	Canidae	-0.229954	-0.341107
Canidae_*Phlaocyon*	NAC	Canidae	1.21182	-0.523815
Canidae_*Psalidocyon*	NAC	Canidae	0.0889423	0.599057
Canidae_*Sunkahetanka*	NAC	Canidae	0.00748426	-0.384004
Canidae_*Tephrocyon*	NAC	Canidae	0.0932822	0.603951
Canidae_*Tomarctus*	NAC	Canidae	0.288235	0.674166
Canidae_*Urocyon*	NAC	Canidae	0.2759	1.29964
Canidae_*Vulpes*	NAC	Canidae	0.275048	1.14045
Felidae_*Felis*_6	NAC	Felidae	-1.63953	-0.999089
Felidae_*Homotherium*	NAC	Felidae	-1.38866	-1.51068
Felidae_*Lynx*	NAC	Felidae	-1.68973	-0.640351

Felidae_*Machairodus*	NAC	Felidae	-1.2199	-0.972257
Felidae_*Nimravides*	NAC	Felidae	-0.930291	-1.17472
Felidae_*Pseudaelurus*	NAC	Felidae	-1.01782	-0.551766
Felidae_*Smilodon*	NAC	Felidae	-1.24064	-1.14458
Hyaenidae_*Chasmaporthetes*	NAC	Hyaenidae	-1.29843	-1.43057
Mephitidae_*Martinogale*	NAC	Mephitidae	-0.2759	1.31445
Mephitidae_*Mephitis*	NAC	Mephitidae	0.57641	-0.704386
Mephitidae_*Spilogale*	NAC	Mephitidae	0.484565	0.236323
Miacoidea_*Bryanictis*	NAC	Miacoidea	0.659758	0.506228
Miacoidea_*Didymictis*	NAC	Miacoidea	0.366128	0.441099
Miacoidea_*Miacis*	NAC	Miacoidea	0.453362	0.639542
Miacoidea_*Oodectes*	NAC	Miacoidea	1.34001	1.04013
Miacoidea_*Palaeogale*	NAC	Miacoidea	-0.941411	0.579535
Miacoidea_*Procynodictis*	NAC	Miacoidea	-0.2655	0.960368
Miacoidea_*Tapocyon*	NAC	Miacoidea	0.329912	-0.468497
Miacoidea_*Uintacyon*	NAC	Miacoidea	0.652401	0.357094
Miacoidea_*Vassacyon*	NAC	Miacoidea	0.943459	0.151791
Miacoidea_*Viverravus*	NAC	Miacoidea	0.122626	1.28936
Miacoidea_*Vulpavus*	NAC	Miacoidea	1.27745	0.609672
Mustelidae_*Brachypsalis*	NAC	Mustelidae	0.00991426	-0.237811
Mustelidae_*Craterogale*	NAC	Mustelidae	0.436843	-0.491756
Mustelidae_*Leptarctus*	NAC	Mephitidae	0.974261	-1.61199
Mustelidae_*Lutravus*	NAC	Mephitidae	0.206009	-0.895219
Mustelidae_*Martes*	NAC	Mustelidae	-0.0588709	0.836905
Mustelidae_*Megalictis*	NAC	Mustelidae	-0.314975	-0.933775

Mustelidae_*Miomustela*	NAC	Mustelidae	-0.567549	1.00086
Mustelidae_*Mionictis*	NAC	Mustelidae	0.645977	-1.03198
Mustelidae_*Mustela*	NAC	Mustelidae	-0.0257205	0.0595775
Mustelidae_*Oligobunis*	NAC	Mustelidae	-0.166766	-0.363508
Mustelidae_*Plesiogulo*	NAC	Mustelidae	-0.13888	-1.49373
Mustelidae_*Plionictis*	NAC	Mustelidae	-0.0959253	0.560949
Mustelidae_*Pliotaxidea*	NAC	Mustelidae	1.24415	-1.21735
Mustelidae_*Potamotherium*	NAC	Mephitidae	0.416914	-1.01216
Mustelidae_*Promartes*	NAC	Mustelidae	0.143108	0.508246
Mustelidae_*Sthenictis*	NAC	Mustelidae	0.0699392	0.176672
Mustelidae_*Taxidea*	NAC	Mustelidae	0.802573	-1.83582
Mustelidae_*Zodiolestes*	NAC	Mustelidae	0.023106	0.457614
Nimravidae_*Dinictis*	NAC	Nimravidae	-0.564599	-0.0253915
Nimravidae_*Hoplophoneus*	NAC	Nimravidae	-0.829078	-0.484449
Nimravidae_*Nimravus*	NAC	Nimravidae	-1.21982	-1.15339
Nimravidae_*Pogonodon*	NAC	Nimravidae	-0.99403	-1.13984
Procyonidae_*Bassariscus*	NAC	Procyonidae	0.882108	0.799776
Procyonidae_*Edaphocyon*	NAC	Procyonidae	2.439	-1.1438
Procyonidae_*Nasua*	NAC	Procyonidae	2.53656	-0.0638593
Procyonidae_*Procyon*	NAC	Procyonidae	2.60924	-0.951222
Ursidae_*Agriotherium*	NAC	Ursidae	1.32831	-2.07716
Ursidae_*Arctodus*	NAC	Ursidae	1.19803	-3.54904
Ursidae_*Cephalogale*	NAC	Ursidae	0.59397	-1.42672
Ursidae_*Hemicyon*	NAC	Ursidae	0.94929	-0.720021
Ursidae_*Ursavus*	NAC	Ursidae	1.60148	-1.60714

Ursidae_*Ursus*	NAC	Ursidae	1.86654	-3.11552
Ailuridae_*Ailurus_fulgens*	MC	Ailuridae	2.2203	-0.63791
Canidae_*Alopex_lagopus*	MC	Canidae	-0.128823	1.15327
Canidae_*Atelocynus_microtis*	MC	Canidae	0.32427	0.93506
Canidae_*Canis_adustus*	MC	Canidae	0.496339	1.31552
Canidae_*Canis_aureus*	MC	Canidae	0.281546	1.02014
Canidae_*Canis_latrans*	MC	Canidae	0.275048	1.14045
Canidae_*Canis_lupus*	MC	Canidae	0.301736	0.419834
Canidae_*Canis_mesomelas*	MC	Canidae	0.275048	1.14045
Canidae_*Canis_rufus*	MC	Canidae	0.368039	0.683844
Canidae_*Canis_simensis*	MC	Canidae	0.281546	1.02014
Canidae_*Cerdocyon_thous*	MC	Canidae	0.262111	1.57689
Canidae_*Chrysocyon_brachyurus*	MC	Canidae	0.454133	0.504589
Canidae_*Cuon_alpinus*	MC	Canidae	-0.345191	0.639838
Canidae_*Lycaon_pictus*	MC	Canidae	-0.177205	0.00487722
Canidae_*Nyctereutes_procyonoides*	MC	Canidae	0.268507	1.35463
Canidae_*Otocyon_megalotis*	MC	Canidae	1.57023	0.658941
Canidae_*Pseudalopex_culpaeus*	MC	Canidae	0.275048	1.14045
Canidae_*Pseudalopex_griseus*	MC	Canidae	0.423551	1.23903
Canidae_*Pseudalopex_gymnocercus*	MC	Canidae	0.262111	1.57689
Canidae_*Pseudalopex_sechurae*	MC	Canidae	0.413627	1.68736
Canidae_*Pseudalopex_vetulus*	MC	Canidae	0.423571	1.57401
Canidae_*Speothos_venaticus*	MC	Canidae	-0.532718	0.464199
Canidae_*Urocyon_cinereoargenteus*	MC	Canidae	0.2759	1.29964
Canidae_*Urocyon_littoralis*	MC	Canidae	0.33565	1.61584

Canidae_*Vulpes_bengalensis*	MC	Canidae	0.413627	1.68736
Canidae_*Vulpes_chama*	MC	Canidae	0.488213	1.75577
Canidae_*Vulpes_rueppelli*	MC	Canidae	0.33565	1.61584
Canidae_*Vulpes_velox*	MC	Canidae	0.262111	1.57689
Canidae_*Vulpes_vulpes*	MC	Canidae	0.262111	1.57689
Canidae_*Vulpes_zerda*	MC	Canidae	0.32322	2.09379
Canidae_*Vulpes_corsac*	MC	Canidae	0.262111	1.57689
Canidae_*Vulpes_pallida*	MC	Canidae	0.413627	1.68736
Eupleridae_*Cryptoprocta_ferox*	MC	Eupleridae	-1.63198	-0.792254
Eupleridae_*Eupleres_goudotii*	MC	Eupleridae	0.835699	1.39937
Eupleridae_*Fossa_fossana*	MC	Eupleridae	0.678107	0.930442
Eupleridae_*Galidia_elegans*	MC	Eupleridae	-0.628914	1.14803
Eupleridae_*Galidictis_fasciata*	MC	Eupleridae	0.200238	0.461422
Eupleridae_*Galidictis_grandidieri*	MC	Eupleridae	0.0282206	-0.162615
Eupleridae_*Salanoia_concolor*	MC	Eupleridae	0.378259	0.584327
Felidae_*Acinonyx_jubatus*	MC	Felidae	-1.52067	-0.942167
Felidae_*Caracal_caracal*	MC	Felidae	-1.57005	-0.500802
Felidae_*Catopuma_badia*	MC	Felidae	-1.73369	-0.227883
Felidae_*Catopuma_temminckii*	MC	Felidae	-1.68055	-0.609681
Felidae_*Felis_bieti*	MC	Felidae	-1.73369	-0.227883
Felidae_*Felis_chaus*	MC	Felidae	-1.64742	-0.463991
Felidae_*Felis_margarita*	MC	Felidae	-1.58793	0.0114134
Felidae_*Felis_nigripes*	MC	Felidae	-1.55543	0.207022
Felidae_*Felis_silvestris*	MC	Felidae	-1.61985	-0.124574
Felidae_*Herpailurus_yaguarondi*	MC	Felidae	-1.61985	-0.124574

Felidae_*Leopardus_pardalis*	MC	Felidae	-1.57005	-0.500802
Felidae_*Leopardus_tigrinus*	MC	Felidae	-1.46991	0.169257
Felidae_*Leopardus_wiedii*	MC	Felidae	-1.61985	-0.124574
Felidae_*Leptailurus_serval*	MC	Felidae	-1.64742	-0.463991
Felidae_*Lynx_canadensis*	MC	Felidae	-1.68055	-0.609681
Felidae_*Lynx_pardinus*	MC	Felidae	-1.68055	-0.609681
Felidae_*Lynx_rufus*	MC	Felidae	-1.79566	-0.723096
Felidae_*Lynx_lynx*	MC	Felidae	-1.67273	-0.558676
Felidae_*Neofelis_nebulosa*	MC	Felidae	-1.68055	-0.609681
Felidae_*Oncifelis_colocolo*	MC	Felidae	-1.65238	-0.307959
Felidae_*Oncifelis_geoffroyi*	MC	Felidae	-1.61985	-0.124574
Felidae_*Oncifelis_guigna*	MC	Felidae	-1.69135	-0.0251184
Felidae_*Otocolobus_manul*	MC	Felidae	-1.61985	-0.124574
Felidae_*Panthera_leo*	MC	Felidae	-1.43193	-1.5443
Felidae_*Panthera_pardus*	MC	Felidae	-1.58053	-1.32836
Felidae_*Panthera_tigris*	MC	Felidae	-1.43193	-1.5443
Felidae_*Panthera_onca*	MC	Felidae	-1.68903	-1.45161
Felidae_*Pardofelis_marmorata*	MC	Felidae	-1.61985	-0.124574
Felidae_*Prionailurus_bengalensis*	MC	Felidae	-1.58793	0.0114134
Felidae_*Prionailurus_planiceps*	MC	Felidae	-1.42086	0.347137
Felidae_*Prionailurus_rubiginosus*	MC	Felidae	-1.78909	0.170161
Felidae_*Prionailurus_viverrinus*	MC	Felidae	-1.57005	-0.500802
Felidae_*Profelis_aurata*	MC	Felidae	-1.64742	-0.463991
Felidae_*Puma_concolor*	MC	Felidae	-1.58053	-1.32836
Felidae_*Uncia_uncia*	MC	Felidae	-1.6616	-1.17323

Herpestidae_*Atilax_paludinosus*	MC	Herpestidae	0.681321	-0.256749
Herpestidae_*Bdeogale_crassicauda*	MC	Herpestidae	1.49606	-0.225068
Herpestidae_*Bdeogale_jacksoni*	MC	Herpestidae	1.60329	-0.103845
Herpestidae_*Bdeogale_nigripes*	MC	Herpestidae	2.14334	-0.705727
Herpestidae_*Crossarchus_alexandri*	MC	Herpestidae	1.38498	0.711419
Herpestidae_*Crossarchus_obscurus*	MC	Herpestidae	1.19053	0.356156
Herpestidae_*Cynictis_penicillata*	MC	Herpestidae	0.42115	0.910454
Herpestidae_*Dologale_dybowskii*	MC	Herpestidae	0.912869	0.604498
Herpestidae_*Galerella_pulverulenta*	MC	Herpestidae	-0.131624	0.57596
Herpestidae_*Galerella_sanguinea*	MC	Herpestidae	-0.442303	1.12937
Herpestidae_*Helogale_hirtula*	MC	Herpestidae	0.951841	0.434675
Herpestidae_*Helogale_parvula*	MC	Herpestidae	1.19344	0.495618
Herpestidae_*Herpestes_ichneumon*	MC	Herpestidae	0.00776258	-0.149973
Herpestidae_*Ichneumia_albicauda*	MC	Herpestidae	1.45048	0.485907
Herpestidae_*Liberiictis_kuhni*	MC	Herpestidae	1.91062	0.162443
Herpestidae_*Mungos_gambianus*	MC	Herpestidae	1.5375	-0.316301
Herpestidae_*Mungos_mungo*	MC	Herpestidae	1.36993	-0.246878
Herpestidae_*Paracynictis_selousi*	MC	Herpestidae	0.870555	0.548532
Herpestidae_*Rhynchogale_melleri*	MC	Herpestidae	1.57202	-0.319008
Herpestidae_*Suricata_suricata*	MC	Herpestidae	1.11217	0.201732
Herpestidae_*Urva_brachyurus*	MC	Herpestidae	-0.0102096	0.20392
Herpestidae_*Urva_edwardsii*	MC	Herpestidae	-0.500341	1.03287
Herpestidae_*Urva_javanicus*	MC	Herpestidae	-0.253961	0.671663
Herpestidae_*Urva_semitorquatus*	MC	Herpestidae	0.00911731	0.0860929
Herpestidae_*Urva_smithii*	MC	Herpestidae	-0.238373	0.00521866

Herpestidae_*Urva_urva*	MC	Herpestidae	0.0217266	0.267576
Herpestidae_*Urva_vitticollis*	MC	Herpestidae	0.315424	0.0804519
Hyaenidae_*Crocuta_crocuta*	MC	Hyaenidae	-1.23703	-1.01858
Hyaenidae_*Hyaena_hyaena*	MC	Hyaenidae	-0.468136	-1.02982
Hyaenidae_*Parahyaena_brunnea*	MC	Hyaenidae	-1.46857	-1.16192
Hyaenidae_*Proteles_cristatus*	MC	Hyaenidae	-0.0134755	-1.75362
Mephitidae_*Conepatus_chinga*	MC	Mephitidae	0.818461	-0.101056
Mephitidae_*Conepatus_humboldtii*	MC	Mephitidae	0.720844	-0.26026
Mephitidae_*Conepatus_leuconotus*	MC	Mephitidae	0.949452	-0.808195
Mephitidae_*Conepatus_mesoleucus*	MC	Mephitidae	0.858978	-0.984521
Mephitidae_*Conepatus_semistriatus*	MC	Mephitidae	0.820027	-0.671811
Mephitidae_*Mephitis_macroura*	MC	Mephitidae	0.515745	-0.0276752
Mephitidae_*Mephitis_mephitis*	MC	Mephitidae	0.57641	-0.704386
Mephitidae_*Mydaus_javanensis*	MC	Mephitidae	1.62336	-0.255827
Mephitidae_*Mydaus_marchei*	MC	Mephitidae	1.39293	-1.18144
Mephitidae_*Spilogale_putorius*	MC	Mephitidae	0.484565	0.236323
Mephitidae_*Spilogale_pygmaea*	MC	Mephitidae	0.447019	0.0141281
Mustelidae_*Amblonyx_cinereus*	MC	Mustelidae	0.36187	-1.11438
Mustelidae_*Aonyx_capensis*	MC	Mustelidae	0.945517	-2.26687
Mustelidae_*Aonyx_congicus*	MC	Mustelidae	0.931822	-2.60166
Mustelidae_*Arctonyx_collaris*	MC	Mustelidae	1.16898	-0.165645
Mustelidae_*Eira_barbara*	MC	Mustelidae	-0.0530006	-0.460068
Mustelidae_*Enhydra_lutris*	MC	Mustelidae	1.40294	-3.32499
Mustelidae_*Galictis_cuja*	MC	Mustelidae	0.252634	-1.02329
Mustelidae_*Galictis_vittata*	MC	Mustelidae	-0.330994	-0.456711

Mustelidae_*Gulo_gulo*	MC	Mustelidae	-0.555674	-0.306695
Mustelidae_*Ictonyx_libyca*	MC	Mustelidae	-0.0179882	0.702749
Mustelidae_*Ictonyx_striatus*	MC	Mustelidae	-0.208692	0.709607
Mustelidae_*Lontra_canadensis*	MC	Mustelidae	0.800047	-1.19823
Mustelidae_*Lontra_felina*	MC	Mustelidae	0.451402	-2.30578
Mustelidae_*Lontra_longicaudis*	MC	Mustelidae	0.426771	-1.32753
Mustelidae_*Lontra_provocax*	MC	Mustelidae	0.0236046	-2.03254
Mustelidae_*Lutra_lutra*	MC	Mustelidae	0.307024	-1.18767
Mustelidae_*Lutra_maculicollis*	MC	Mustelidae	0.300574	-0.913063
Mustelidae_*Lutra_sumatrana*	MC	Mustelidae	0.235536	-0.29335
Mustelidae_*Lutrogale_perspicillata*	MC	Mustelidae	0.835591	-1.76005
Mustelidae_*Martes_americana*	MC	Mustelidae	-0.0588709	0.836905
Mustelidae_*Martes_flavigula*	MC	Mustelidae	-0.183006	0.241856
Mustelidae_*Martes_foina*	MC	Mustelidae	-0.710925	0.814083
Mustelidae_*Martes_martes*	MC	Mustelidae	-0.100964	0.740118
Mustelidae_*Martes_melampus*	MC	Mustelidae	-0.134983	0.63418
Mustelidae_*Martes_pennanti*	MC	Mustelidae	-0.390849	0.724097
Mustelidae_*Martes_zibellina*	MC	Mustelidae	0.0255451	0.734888
Mustelidae_*Meles_meles*	MC	Mustelidae	0.926959	-1.22131
Mustelidae_*Mellivora_capensis*	MC	Mustelidae	-0.837686	-0.590339
Mustelidae_*Melogale_everetti*	MC	Mustelidae	0.184456	0.0363049
Mustelidae_*Melogale_moschata*	MC	Mustelidae	0.295329	0.960904
Mustelidae_*Melogale_orientalis*	MC	Mustelidae	0.34996	0.258231
Mustelidae_*Melogale_personata*	MC	Mustelidae	0.509902	-0.341463
Mustelidae_*Mustela_africana*	MC	Mustelidae	-0.601739	0.83357

Mustelidae_*Mustela_altaica*	MC	Mustelidae	-0.561291	1.17242
Mustelidae_*Mustela_erminea*	MC	Mustelidae	-0.601739	0.83357
Mustelidae_*Mustela_eversmannii*	MC	Mustelidae	-0.638199	0.838802
Mustelidae_*Mustela_felipei*	MC	Mustelidae	-0.826888	0.736447
Mustelidae_*Mustela_frenata*	MC	Mustelidae	-0.280931	0.463998
Mustelidae_*Mustela_kathiah*	MC	Mustelidae	-0.0585943	0.908642
Mustelidae_*Mustela_lutreola*	MC	Mustelidae	-0.461356	0.994804
Mustelidae_*Mustela_lutreolina*	MC	Mustelidae	-0.595267	1.07628
Mustelidae_*Mustela_nigripes*	MC	Mustelidae	-0.635382	0.568918
Mustelidae_*Mustela_nivalis*	MC	Mustelidae	-0.57326	0.993721
Mustelidae_*Mustela_nudipes*	MC	Mustelidae	-0.388451	0.619722
Mustelidae_*Mustela_putorius*	MC	Mustelidae	-0.182704	0.568052
Mustelidae_*Mustela_sibirica*	MC	Mustelidae	-0.536317	0.929608
Mustelidae_*Mustela_vison*	MC	Mustelidae	0.0246016	0.112025
Mustelidae_*Poecilogale_albinucha*	MC	Mustelidae	-1.00872	0.538913
Mustelidae_*Pteronura_brasiliensis*	MC	Mustelidae	0.528189	-1.88155
Mustelidae_*Taxidea_taxus*	MC	Mustelidae	0.485037	-1.49844
Mustelidae_*Vormela_peregusna*	MC	Mustelidae	-0.424386	0.245373
Nandiniidae_*Nandinia_binotata*	MC	Nandiniidae	-0.278663	0.620818
Prionodontidae_*Prionodon_linsang*	MC	Prionodontidae	-0.420345	1.54619
Prionodontidae_*Prionodon_pardicolor*	MC	Prionodontidae	-0.632604	1.37827
Procyonidae_*Bassaricyon_alleni*	MC	Procyonidae	2.51642	0.455158
Procyonidae_*Bassaricyon_gabbi*	MC	Procyonidae	2.92225	0.0839097
Procyonidae_*Bassaricyon_pauli*	MC	Procyonidae	2.92225	0.0839097
Procyonidae_*Bassariscus_astutus*	MC	Procyonidae	0.298642	1.55571

Procyonidae_*Bassariscus_sumichrasti*	MC	Procyonidae	1.04849	0.442047
Procyonidae_*Nasua_narica*	MC	Procyonidae	2.53656	-0.0638593
Procyonidae_*Nasua_nasua*	MC	Procyonidae	2.57046	0.272369
Procyonidae_*Nasuella_olivacea*	MC	Procyonidae	2.32306	0.756777
Procyonidae_*Potos_flavus*	MC	Procyonidae	2.9091	-0.997737
Procyonidae_*Procyon_cancrivorus*	MC	Procyonidae	2.29247	-0.559018
Procyonidae_*Procyon_gloveralleni*	MC	Procyonidae	2.37061	-0.249189
Procyonidae_*Procyon_insularis*	MC	Procyonidae	2.22381	-0.689768
Procyonidae_*Procyon_lotor*	MC	Procyonidae	2.27783	-0.505633
Procyonidae_*Procyon_maynardi*	MC	Procyonidae	2.37061	-0.249189
Procyonidae_*Procyon_pygmaeus*	MC	Procyonidae	2.32148	-0.418637
Ursidae_*Ailuropoda_melanoleuca*	MC	Ursidae	2.10334	-2.76305
Ursidae_*Helarctos_malayanus*	MC	Ursidae	1.75727	-1.59599
Ursidae_*Melursus_ursinus*	MC	Ursidae	1.46395	-2.39588
Ursidae_*Tremarctos_ornatus*	MC	Ursidae	1.55623	-2.82143
Ursidae_*Ursus_americanus*	MC	Ursidae	1.66652	-2.78371
Ursidae_*Ursus_arctos*	MC	Ursidae	1.74935	-3.53154
Ursidae_*Ursus_thibetanus*	MC	Ursidae	1.47148	-2.90162
Ursidae_*Ursus_maritimus*	MC	Ursidae	1.32726	-3.56162
Viverridae_*Arctictis_binturong*	MC	Viverridae	1.21434	-0.784637
Viverridae_*Arctogalidia_trivirgata*	MC	Viverridae	2.00102	-0.578816
Viverridae_*Civettictis_civetta*	MC	Viverridae	0.897455	0.039974
Viverridae_*Cynogale_bennettii*	MC	Viverridae	2.02645	0.431446
Viverridae_*Diplogale_hosei*	MC	Viverridae	1.18359	0.740355
Viverridae_*Genetta_abyssinica*	MC	Viverridae	-0.0334365	1.34489

Viverridae_*Genetta_angolensis*	MC	Viverridae	-0.103801	0.535014
Viverridae_*Genetta_genetta*	MC	Viverridae	0.613036	0.506599
Viverridae_*Genetta_maculata*	MC	Viverridae	-0.0952791	0.985976
Viverridae_*Genetta_servalina*	MC	Viverridae	-0.00147229	1.0776
Viverridae_*Genetta_thierryi*	MC	Viverridae	0.00778907	0.863205
Viverridae_*Genetta_tigrina*	MC	Viverridae	-0.335312	0.432824
Viverridae_*Genetta_victoriae*	MC	Viverridae	0.0420126	0.56585
Viverridae_*Hemigalus_derbyanus*	MC	Viverridae	1.93912	0.723566
Viverridae_*Paguma_larvata*	MC	Viverridae	0.920388	-0.302233
Viverridae_*Paradoxurus_hermaphroditus*	MC	Viverridae	1.22316	0.0235851
Viverridae_*Paradoxurus_jerdoni*	MC	Viverridae	1.51601	0.0889113
Viverridae_*Paradoxurus_zeylonensis*	MC	Viverridae	1.41094	-0.00364223
Viverridae_*Poiana_richardsonii*	MC	Viverridae	-0.333601	0.917126
Viverridae_*Viverra_megaspila*	MC	Viverridae	0.574069	0.599924
Viverridae_*Viverra_tangalunga*	MC	Viverridae	0.351898	1.49059
Viverridae_*Viverra_zibetha*	MC	Viverridae	0.661989	0.558093
Viverridae_*Viverricula_indica*	MC	Viverridae	0.501663	0.824964

Table S12. Area of morphospace occupied in figure 2 and mean distance between taxa for selected data sets and modern carnivoran families. Abbreviation: N, number of taxa.

Group	Area	Mean Distance	N
Canidae	2.1477	0.097288	31
Eupleridae	2.4035	0.36743	7
Felidae	0.60112	0.034568	35
Herpestidae	2.7066	0.1752	27
Hyaenidae	0.4485	0.24325	4
Malaysia	11.409	0.24205	27
Mephitidae	1.0207	0.18964	11
Modern Carnivora (all)	17.468	0.12007	216
Modern South American Carnivora	13.681	0.25211	40
Mustelidae	6.0645	0.19091	51
North American Carnivoramorpha	13.93	0.18299	95
Procyonidae	2.5734	0.14675	15
Santa Cruz, Argentina (early Miocene)	0.74619	0.1973	11
Serengeti National Park, Tanzania	5.5325	0.29176	24
Sparassodonta	2.0765	0.13685	41
Ursidae	0.86421	0.16873	8
Viverridae	3.5053	0.21927	23

Yellowstone National Park, U.S.A.	11.566		17

References

[1] Forasiepi AM. 2009 Osteology of *Arctodictis sinclairi* (Mammalia, Metatheria, Sparassodonta) and phylogeny of Cenozoic metatherian carnivores from South America. *Mongr. Mus. Arg. Cien. Nat.* **6**, 1-174.

[2] Zimicz N. 2014 Avoiding competition: the ecological history of late Cenozoic metatherian carnivores in South America. *J. Mammal. Evol.* **21**, 383-393. (doi:10.1007/s10914-014-9255-8).

[3] Prevosti FJ, Forasiepi A & Zimicz N. 2013 The evolution of the Cenozoic terrestrial mammal guild in South America: competition or replacement? *J. Mammal. Evol.* **20**, 3-21. (doi:10.1007/s10914-011-9175-9).

[4] Wroe S, Argot C & Dickman C. 2004 On the rarity of big, fierce carnivores and primacy of isolation and area: tracking large mammalian carnivore diversity on two isolated continents. *P. Roy. Soc. B-Biol. Sci.* **271**, 1203-1211.

[5] Prevosti FJ, Forasiepi AM, Ercoli MD & Turazzini GF. 2012 Paleoecology of the mammalian carnivores (Metatheria, Sparassodonta) of the Santa Cruz Formation (late Early Miocene). In *Early Miocene paleobiology in Patagonia: high-latitude paleocommunities of the Santa Cruz Formation* (eds Vizcaíno SF, Kay RF & Bargo MS), pp. 173-193. Cambridge, Cambridge University Press.

[6] Vizcaíno SF, Bargo MS, Kay RF, Fariña RA, Di Giacomo M, Perry JMG, Prevosti FJ, Toledo N, Cassini GH & Fernicola JC. 2010 A baseline paleoecological study for the Santa Cruz Formation (late-early Miocene) at the Atlantic coast of Patagonia, Argentina. *Palaeogeogr. Palaeoclimatol. Palaeoecol.* **292**, 507-519.

[7] Ercoli MD & Prevosti FJ. 2011 Estimación de masa de las especies de Sparassodonta (Mammalia, Metatheria) de la edad santacrucense (Mioceno temprano) a partir del tamaño del centroide de los elementos apendiculares: inferencias paleoecológicas. *Ameghiniana* **48**, 462-479.

[8] Forasiepi AM, Judith Babot M & Zimicz N. 2015 *Australohyaena antiqua* (Mammalia, Metatheria, Sparassodonta), a large predator from the Late Oligocene of Patagonia. *J. Syst. Palaeontol.* **13**, 503-525. (doi:10.1080/14772019.2014.926403).

[9] Argot C. 2003b Functional adaptations of the postcranial skeleton of two Miocene borhyaenoids (Mammalia, Metatheria), *Borhyaena* and *Prothylacinus*, from South America. *Palaeontology* **46**, 1213-1267.

[10] Argot C & Babot J. 2011 Postcranial morphology, functional adaptations and palaeobiology of *Callistoe vincei*, a predaceous metatherian from the Eocene of Salta, north-western Argentina. *Palaeontology* **54**, 447-480. (doi:10.1111/j.1475-4983.2011.01036.x).

[11] Argot C. 2003c Postcranial functional adaptations in the South American Miocene borhyaenoids (Mammalia, Metatheria): *Cladosictis*, *Pseudonotictis* and *Sipalocyon*. *Alcheringa* **27**, 303-356.

[12] Wroe S, Myers TJ, Wells RT & Gillespie A. 1999 Estimating the weight of the Pleistocene marsupial lion, *Thylacoleo carnifex* (Thylacoleonidae : Marsupialia): implications for the ecomorphology of a marsupial super-predator and

hypotheses of impoverishment of Australian marsupial carnivore faunas. *Aust. J. Zool.* **47**, 489-498.

[13] Marshall LG. 1978 Evolution of the Borhyaenidae, extinct South American predaceous marsupials. *U. Calif. Pub. Geol. Sci.* **117**, 1-89.

[14] Myers TJ. 2001 Prediction of marsupial body mass. *Aust. J. Zool.* **49**, 99-118.

[15] Goin FJ. 1997 New clues for understanding Neogene marsupial radiations. In *Vertebrate paleontology in the Neotropics: the Miocene fauna of La Venta, Colombia* (eds Kay RF, Madden RH, Cifelli RL & Flynn JJ), pp. 187-206. Washington, DC, United States, Smithsonian Institution Press.

[16] Riggs ES. 1934 A new marsupial saber-tooth from the Pliocene of Argentina and its relationships to other South American predacious marsupials. *T. Am. Philos. Soc.* **24**, 1-32.

[17] Simpson GG. 1970 Mammals from the early Cenozoic of Chubut, Argentina. *Breviora*, 1-13.

[18] Jerison HJ. 1971 Quantitative analysis of the evolution of the camelid brain. *Am Nat* **105**, 227-239.

[19] Silva M & Downing JA. 1995 *CRC Handbook of mammalian body masses*. New York, CRC Press; 359 p.

[20] Babot MJ, Powell JE & Muizon Cd. 2002 *Callistoe vincei*, a new Proborhyaenidae (Borhyaenoidea, Metatheria, Mammalia) from the Early Eocene of Argentina. *Geobios* **35**, 615-629.

[21] Forasiepi AM, Martinelli AG & Goin FJ. 2007 Revisión taxonómica de *Parahyaenodon argentinus* Ameghino y sus implicancias en el conocimiento de los grandes mamíferos carnívoros del Mio-Plioceno de América de Sur. *Ameghiniana* **44**, 143-159.

[22] Reig OA. 1957 Nota previa sobre los marsupiales de la formación Chasicó. *Ameghiniana* **1**, 27-31.

[23] Marshall LG. 1981 Review of the Hathlyacyninae, an extinct subfamily of South American "dog-like" marsupials. *Fieldiana: Geology (New Series)* **7**, 1-120.

[24] Sinclair WJ. 1906 Mammalia of the Santa Cruz Beds. Volume IV, Paleontology. Part III, Marsupialia. In *Reports of the Princeton University expeditions to Patagonia, 1896-1899* (ed Scott WB), pp. 333-460. Stuttgart, Princeton University, E. Schweizerbart'sche Verlagshandlung (E. Nägele).

[25] Marshall LG. 1977 A new species of *Lycopsis* (Borhyaenidae: Marsupialia) from La Venta Fauna (late Miocene) of Colombia, South America. *J. Paleontol.* **51**, 633-642.

[26] Shockey BJ & Anaya F. 2008 Postcranial osteology of mammals from Salla, Bolivia (late Oligocene): form, function, and phylogenetic implications. In *Mammalian evolutionary morphology: a tribute to Frederick S. Szalay* (eds Sargis EJ & Dagosto M), pp. 135-157. New York, Springer.

[27] Tejedor MF, Goin FJ, Gelfo JN, López G, Bond M, Carlini AA, Scillato-Yané GJ, Woodburne MO, Chornogubsky L, Aragón E, et al. 2009 New early Eocene

mammalian fauna from western Patagonia, Argentina. *Am. Mus. Novit.* **3638**, 1-43.

[28] Marshall LG. 1976 New didelphine Marsupials from the La Venta Fauna (Miocene) of Colombia, South America. *J. Paleontol.* **50**, 402-418.

[29] Simpson GG. 1948 The beginning of the age of mammals in South America. Part I. *B. Am. Mus. Nat. Hist.* **91**, 1-232.

[30] Villarroel C & Marshall LG. 1982 Geology of the Deseadan (early Oligocene) age "Estratos Salla" in the Salla-Luribay Basin, Bolivia, with description of new Marsupialia. *Geobios, mémoire spécial* **6**, 201-211.

[31] Forasiepi AM & Carlini AA. 2010 A new thylacosmilid (Mammalia, Metatheria, Sparassodonta) from the Miocene of Patagonia, Argentina. *Zootaxa* **2552**, 55-68.

[32] Patterson B & Marshall LG. 1978 The Deseadan, early Oligocene, Marsupialia of South America. *Fieldiana Geology* **41**, 37-100.

[33] Marshall LG. 1976 A new borhyaenid (Marsupialia, Boryaeninae) from the Arroyo Chasicó Formation (lower Pliocene), Buenos Aires Province, Argentina. *Ameghiniana* **13**, 289-299.

[34] Petter G & Hoffstetter R. 1983 Les marsupiaux du Déséadien (Oligocène inférieur) de Salla (Bolivie). *Ann. Paleontol.* **69**, 175-234.

[35] Forasiepi AM, Sánchez-Villagra MR, Goin FJ, Takai M, Shigehara N & Kay RF. 2006 A new species of Hathliacynidae (Metatheria, Sparassodonta) from the middle Miocene of Quebrada Honda, Bolivia. *J. Vertebr. Paleontol.* **26**, 670-684.

[36] Powell JE, Babot MJ, García López DA, Deraco MV & Herrera C. 2011 Eocene vertebrates of northwestern Argentina: annotated list. In *Cenozoic geology of the central Andes of Argentina* (eds Salfity JA & Marquillas RA), pp. 349-370. Salta, SCS Publisher.

[37] Suarez C, Forasiepi AM, Goin FJ & Jaramillo C. 2015 Insights into the Neotropics prior to the Great American Biotic Interchange: new evidence of mammalian predators from the Miocene of northern Colombia. *J. Vertebr. Paleontol.* **36**, e1029581. (doi:10.1080/02724634.2015.1029581).

[38] Goin FJ, Abello A, Bellosi E, Kay R, Madden R & Carlini A. 2007 Los Metatheria sudamericanos de comienzos del Neógeno (Mioceno Temprano, Edad-mamífero Colhuehuapense). Parte I: Introducción, Didelphimorphia y Sparassodonta. *Ameghiniana* **44**, 29-71.

[39] Goin FJ & Candela A. 2004 New Paleogene marsupials from the Amazon Basin of eastern Perú. *Sci. Ser., Nat. Hist. Mus. Los Angelas Co.* **40**, 15-60.

[40] Engelman RK & Croft DA. 2014 A new species of small-bodied sparassodont (Mammalia, Metatheria) from the middle Miocene locality of Quebrada Honda, Bolivia. *J. Vertebr. Paleontol.* **34**, 672-688.

[41] Goin FJ, Abello MA & Chornogubsky L. 2010 Middle Tertiary marsupials from central Patagonia (early Oligocene of Gran Barranca): understanding South America's *Grande Coupure*. In *The paleontology of Gran Barranca. Evolution and environmental change through the middle Cenozoic of Patagonia* (eds Madden

RH, Carlini AA, Vucetich MG & Kay RF), pp. 69-105. Cambridge, Cambridge University Press.

[42] Babot MJ & Ortiz PE. 2008 Primer registro de Borhyaenoidea (Mammalia, Metatheria, Sparassodonta) en la provincia de Tucumán (Formación India Muerta, Grupo Choromoro; Mioceno tardío). *Acta Geol. LilloanA* **21**, 34-48.

[43] Marshall LG. 1979 Review of the Prothylacyninae, an extinct subfamily of South American "dog-like" marsupials. *Fieldiana: Geology (New Series)* **3**, 1-50.

[44] Sedor FA, Oliveira ÉV, Silva DD, Fernandes LA, Cunha RF, Ribeiro AM & Dias EV. 2017 A new South American Paleogene land mammal fauna, Guabirotuba Formation (southern Brazil). *J. Mammal. Evol.* **24**, 39-55. (doi:10.1007/s10914-016-9364-7).

[45] Hoffstetter R & Petter G. 1983 *Paraborhyaena boliviana* et *Andinogale sallensis*, deux Marsupiaux (Borhyaenidae) nouveaux de Déséadien (Oligocène inférieur) de Salla (Bolivie). *CR. Acad. Sci. III* **296**, 143-146.

[46] Goin FJ, Palma RM, Pascual R & Powell JE. 1986 Persisténcia de un primitivo Borhyaenidae (Mammalia, Marsupialia) en el Eoceno temprano de Salta (Fm. Lumbrera, Argentina). Aspectos geológicos y paleoambientales relacionados. *Ameghiniana* **23**, 47-56.

[47] Martin GM & Tejedor MF. 2007 Nueva especie de *Pseudonotictis* Ameghino (Metatheria, Sparassodonta, Hathliacynidae) del Mioceno medio de Chubut noroccidental, Argentina. *Ameghiniana* **44**, 747-750.

[48] Goin FJ & Pascual R. 1987 News on the biology and taxonomy of the marsupials Thylacosmilidae (late Tertiary of Argentina). *An. Acad. Naci. Cien. Exact. Fis. Nat. Buenos Aires* **39**, 219-246.

[49] Goin FJ & Pardiñas UFJ. 1996 Revisión de las especies del género *Hyperdidelphys* Ameghino, 1904 (Mammalia, Marsupialia, Didelphidae). Su significación filogenética, estratigráfica y adaptativa en el Neógeno del Cono Sur sudamericano. *Estud. Geol.-Madrid* **52**, 327-359.

[50] Reig OA & Simpson GG. 1972 *Sparassocynus* (Marsupialia Didelphidae), a peculiar mammal from the late Cenozoic of Argentina. *J. Zool* **167**, 511-539.

[51] Reig OA. 1952 Descripción previa de nuevos ungulados y marsupiales fósiles del Plioceno y del eocuartario argentinos. *Rev. Mus. Mun. Cienc. Nat. Trad. Mar del Plata* **1**, 119-129.

[52] Wesley-Hunt GD. 2005 The morphological diversification of carnivores in North America. *Paleobiology* **31**, 35-55.

[53] Werdelin L & Wesley-Hunt GD. 2010 The biogeography of carnivore evolution. In *Carnivoran evolution: new views on phylogeny, form, and function* (eds Goswami A & Friscia A), pp. 225-245. Cambridge, Cambridge University Press.

[54] Van Valkenburgh B. 1989 Carnivore dental adaptations and diet: a study of trophic diversity within guilds. In *Carnivore behavior, ecology, and evolution* (ed Gittleman JL), pp. 410-436. Ithaca, New York, Cornell University Press.

[55] Werdelin L. 1987 Jaw geometry and molar morphology in marsupial carnivores; analysis of a constraint and its macroevolutionary consequences. *Paleobiology* **13**, 342-350.

[56] Jones ME. 2003 Convergence in ecomorphology and guild structure among marsupial and placental carnivores. In *Predators with pouches: the biology of marsupial carnivores* (eds Jones M, Dickman C & Archer M), pp. 285-296. Collingwood, CSIRO Publishing.

[57] Cifelli R & Muizon Cd. 1997 Dentition and jaw of *Kokopellia juddi*, a primitive marsupial or near marsupial from the medial Cretaceous of Utah. *J. Mammal. Evol.* **4**, 241-258. (doi:10.1023/a:1027394430433).

[58] Clemens WAJ. 1966 Fossil Mammals of the type Lance Formation Wyoming Part II. Marsupialia. *U. Calif. Pub. Geol. Sci.* **62**, 1-122.

[59] Godthelp H, Wroe S & Archer M. 1999 A new marsupial from the early Eocene Tingamarra Local Fauna of Murgon, Southeastern Queensland: a prototypical Australian marsupial? *J. Mammal. Evol.* **6**, 289-313.

[60] Korth WW. 1994 Middle Tertiary marsupials (Mammalia) from North America. *J. Paleontol.* **68**, 376-397.

[61] Reig OA, Krisch JAW & Marshall LG. 1987 Systematic relationships of the living and Neocenozoic American "opossum-like" marsupials (suborder Didelphimorpha) with comments on the classification of these and of the Cretaceous and Paleogene New World and European metatherians. In *Possums and opossums: studies in evolution, vol. I* (ed Archer M), pp. 1-89. Chipping

Norton, Australia, Surrey Beatty and Sons in association with the Royal Zoological Society of New South Wales.

[62] Flynn JJ & Swisher CC, III. 1995 Cenozoic South American Land Mammal Ages: correlation to global geochronologies. In *Geochronology, time scales, and global stratigraphic correlation* (eds Berggren WA, Kent DV, Aubry M-P & Hardenbol J), pp. 317-333, SEPM (Society for Sedimentary Geology) Special Publication No. 54.

[63] Gelfo JN, Goin FJ, Woodburne MO & Muizon Cd. 2009 Biochronological relationships of the earliest South American Paleogene mammalian faunas. *Palaeontology* **52**, 251-269.

[64] Krause JM, Clyde WC, Ibañez-Mejía M, Schmitz MD, Barnum T, Bellosi ES & Wilf P. 2017 New age constraints for early Paleogene strata of central Patagonia, Argentina: Implications for the timing of South American Land Mammal Ages. *Geol. Soc. Am. Bull.* **129**, 886-903. (doi:10.1130/b31561.1).

[65] López GM. 2010 Divisaderan: Land Mammal Age or local fauna? In *The paleontology of Gran Barranca. Evolution and environmental change through the middle Cenozoic of Patagonia* (eds Madden RH, Carlini AA, Vucetich MG & Kay RF), pp. 410-420. Cambridge, Cambridge University Press.

[66] Ré GH, Bellosi ES, Heizler M, Vilas JF, Madden RH, Carlini AA, Kay RF & Vucetich MG. 2010 A geochronology for the Sarmiento Formation at Gran Barranca. In *The paleontology of Gran Barranca. Evolution and environmental change*

through the middle Cenozoic of Patagonia (eds Madden RH, Carlini AA, Vucetich MG & Kay RF), pp. 46-58. Cambridge, Cambridge University Press.

[67] Dunn RE, Madden RH, Kohn MJ, Schmitz MD, Strömberg CAE, Carlini AA, Ré GH & Crowley J. 2013 A new chronology for middle Eocene-early Miocene South American Land Mammal Ages. *Geol. Soc. Am. Bull.* **125**, 539-555. (doi:10.1130/b30660.1).

[68] Campbell KE, Jr. (ed) 2004 The Paleogene Mammalian Fauna of Santa Rosa, Amazonian Peru. Los Angeles, Natural History Museum of Los Angeles County; 163 p.

[69] Bond M, Tejedor MF, Campbell Jr KE, Chornogubsky L, Novo N & Goin F. 2015 Eocene primates of South America and the African origins of New World monkeys. *Nature* **520**, 538-541. (doi:10.1038/nature14120).

[70] Madden RH, Guerrero J, Kay RF, Flynn JJ, Swisher CC, III & Walton AH. 1997 The Laventan Stage and Age. In *Vertebrate paleontology in the Neotropics: The Miocene fauna of La Venta, Colombia* (eds Kay RF, Madden RH, Cifelli RL & Flynn JJ), pp. 499-519. Washington, D.C., Smithsonian Institution Press.

[71] Croft DA, Anaya F, Auerbach D, Garzione C & MacFadden BJ. 2009 New data on Miocene Neotropical provinciality from Cerdas, Bolivia. *J. Mammal. Evol.* **16**, 175-198.

[72] Hunt RM, Jr. 1998 Amphicyonidae. In *Evolution of Tertiary mammals of North America: volume 1, Terrestrial carnivores, ungulates, and ungulatelike mammals*

(eds Janis CM, Scott KM & Jacobs LL), pp. 196-227. Cambridge, Cambridge University Press.

[73] Prothero DR & Emry RJ. 2004 The Chadronian, Orellan, and Whitneyan North American Land Mammal Ages. In *Late Cretaceous and Cenozoic mammals of North America* (ed Woodburne MO), pp. 156-168. New York, Columbia University Press.

[74] Van Valkenburgh B. 1994 Ecomorphological analysis of fossil vertebrates and their paleocommunities. In *Ecological morphology. Integrative organismal biology* (eds Wainwright PC & Reilly SM), pp. 140-166. Chicago, University of Chicago Press.

[75] Boardman GS & Hunt RM. 2015 New material and evaluation of the chronostratigraphic position of *Daphoenictis tedfordi* (Mammalia, Carnivora, Amphicyonidae), a cat-like carnivoran from the latest Eocene of northwestern Nebraska, USA. *Palaeontol. Electron.* **18**, 10. (doi:10.26879/508).

[76] Bryant HN. 1996 Nimravidae. In *The terrestrial Eocene-Oligocene transition in North America* (eds Prothero DR & Emry RJ), pp. 453-475. Cambridge, Cambridge University Press.

[77] Barrett PZ. 2016 Taxonomic and systematic revisions to the North American Nimravidae (Mammalia, Carnivora). *PeerJ* **4**, e1658. (doi:10.7717/peerj.1658).

[78] Prothero DR & Whittlesey KE. 1998 Magnetic stratigraphy and biostratigraphy of the Orellan and Whitneyan land-mammal “ages” in the White River Group. In *Depositional environments, lithostratigraphy, and biostratigraphy of the White River and Arikaree groups (Late Eocene to Early Miocene, North America)* (eds

Terry DO, Jr., LaGarry HE & Hunt RM, Jr.), pp. 39-61. Boulder, Colorado, Geological Society of America Special Paper 325.

[79] Croft DA. 2006 Do marsupials make good predators? Insights from predator-prey diversity ratios. *Evol. Ecol. Res.* **8**, 1193-1214.

[80] Nowak RM (ed) 1999 Walker's mammals of the world, sixth ed. Baltimore, Johns Hopkins University Press; li + 1936 pp. p.

[81] Zhou Y, Chen W, Kaneko Y, Newman C, Liao Z, Zhu X, Buesching CD, Xie Z & Macdonald DW. 2015 Seasonal dietary shifts and food resource exploitation by the hog badger (*Arctonyx collaris*) in a Chinese subtropical forest. *Eur. J. Wildlife Res.* **61**, 125-133. (doi:10.1007/s10344-014-0881-5).

[82] Friscia AR, Van Valkenburgh B & Biknevicius AR. 2007 An ecomorphological analysis of extant small carnivorans. *J. Zool* **272**, 82-100.

[83] Baskin JA & Tedford RH. 1996 Small arctoid and feliform carnivorans. In *The terrestrial Eocene-Oligocene transition in North America* (eds Prothero DR & Emry RJ), pp. 486-497. Cambridge, Cambridge University Press.

[84] Wang X. 1994 Phylogenetic systematics of the Hesperocyoninae (Carnivora, Canidae). *B. Am. Mus. Nat. Hist.* **221**, 1-207.

[85] Tedford RH, Wang X & Taylor BE. 2009 Phylogenetic systematics of the North American fossil Caninae (Carnivora, Canidae). *B. Am. Mus. Nat. Hist.* **325**, 1-218.

[86] Wang X, Tedford RH & Taylor BE. 1999 Phylogenetic systematics of the Borophaginae (Carnivora: Canidae). *B. Am. Mus. Nat. Hist.* **243**, 1-391.

[87] Hunt RM, Jr. 2001 Small Oligocene amphicyonids from North America (*Paradaphoenus*, Mammalia, Carnivora). *Am. Mus. Novit.*, 1-20.

[88] Ercoli MD, Prevosti FJ & Forasiepi AM. 2014 The structure of the mammalian predator guild in the Santa Cruz Formation (late early Miocene). *J. Mammal. Evol.* **21**, 369-381. (doi:10.1007/s10914-013-9243-4).

[89] Marshall LG. 1977 Evolution of the carnivorous adaptive zone in South America. In *Major patterns in vertebrate evolution* (eds Hecht MK, Goody PC & Hecht BM), pp. 709-721. New York, Plenum Press.

[90] Degrange FJ, Noriega JI & Areta JI. 2012 Diversity and paleobiology of Santacrucian birds. In *Early Miocene paleobiology in Patagonia: high-latitude paleocommunities of the Santa Cruz Formation* (eds Vizcaíno SF, Kay RF & Bargo MS), pp. 138-155. Cambridge, Cambridge University Press.

[91] www.Serengeti.org. Accessed 2017-06-15

[92] Wang X & Tedford RH. 1996 Canidae. In *The terrestrial Eocene-Oligocene transition in North America* (eds Prothero DR & Emry RJ), pp. 433-452. Cambridge, Cambridge University Press.

[93] Van Valkenburgh B, Wang X & Damuth J. 2004 Cope's Rule, hypercarnivory, and extinction in North American canids. *Science* **306**, 101-104.
